# Genomic Organization, Tissue Distribution and Functional Characterization of the Rat *Pate* Gene Cluster

**DOI:** 10.1371/journal.pone.0032633

**Published:** 2012-03-30

**Authors:** Angireddy Rajesh, Suresh Yenugu

**Affiliations:** Department of Animal Sciences, University of Hyderabad, Hyderabad, India; University of South Florida College of Medicine, United States of America

## Abstract

The cysteine rich prostate and testis expressed (*Pate*) proteins identified till date are thought to resemble the three fingered protein/urokinase-type plasminogen activator receptor proteins. In this study, for the first time, we report the identification, cloning and characterization of rat *Pate* gene cluster and also determine the expression pattern. The rat *Pate* genes are clustered on chromosome 8 and their predicted proteins retained the ten cysteine signature characteristic to TFP/Ly-6 protein family. PATE and PATE-F three dimensional protein structure was found to be similar to that of the toxin bucandin. Though *Pate* gene expression is thought to be prostate and testis specific, we observed that rat *Pate* genes are also expressed in seminal vesicle and epididymis and in tissues beyond the male reproductive tract. In the developing rats (20–60 day old), expression of *Pate* genes seem to be androgen dependent in the epididymis and testis. In the adult rat, androgen ablation resulted in down regulation of the majority of *Pate* genes in the epididymides. PATE and PATE-F proteins were found to be expressed abundantly in the male reproductive tract of rats and on the sperm. Recombinant PATE protein exhibited potent antibacterial activity, whereas PATE-F did not exhibit any antibacterial activity. *Pate* expression was induced in the epididymides when challenged with LPS. Based on our results, we conclude that rat PATE proteins may contribute to the reproductive and defense functions.

## Introduction

Spermatogenesis and sperm maturation occur in the testis and epididymis respectively. In the testis, a number of morphological, molecular and biochemical events allow the differentiation to spermatids [1]. Spermatozoa that leave the gonads are immature, non-motile and lack fertilizing ability and undergo post-gonadal differentiation in the epididymis. Their passage through the epididymis allows interaction with a wide variety of epididymal secreted proteins resulting in acquisition of motility and fertilizing ability [1]. Besides maturation in the epididymis, factors present in the secretions of the prostate and seminal vesicles are also thought to be involved in production of functional spermatozoa [2], [3], [4]. Epididymal and seminal vesicle fluid consists of a wide variety of proteins [5] which includes defensins [6], [7], lipocalins [8], cathelicidins [9], members of the sperm associated antigen 11 family [10], protease inhibitors [11], [12], [13], inhibitors of complement lysis [14], [15], lysozymes [16], [17] and the cysteine rich proteins such as CRISPs [18] and members of the PATE family [19], [20], [21], [22].

*Pate* gene family members identified in mouse and humans [19], [20], [21], [22] are located on chromosomes 11 and 9 respectively. The PATE proteins contain 10 cysteine residues and display an interesting feature wherein the cysteine at the C-terminal end is placed next to an aspargine to form a cysteine-aspargine (CN) dipeptide sequence [19]. The cysteines of PATE proteins form two motifs (C[XX]C[X7–8]C[X6]C[X7–8]C and C[X3]C[X15–16]CC[X4–5]CN. *Pate* genes in both mouse and humans are located closer to acrosomal vesicle protein 1 (*ACRV1*) gene, which encodes a protein that also contains 10 cysteine residues. Further, a characteristic feature of PATE proteins is that the distribution of the cysteine array resembles to that found in the three-fingered proteins (TFPs) [23], [24], uPAR and murine Ly-6 GPI anchored proteins [24], and activin receptors [25]. The functional roles of PATE proteins are not well characterized. Recent reports indicate their neuromodulatory activity [21] and inhibition of calcium uptake in the spermatozoa [26]. Cysteine rich proteins such as eppin and members of the WFDC family display potent antimicrobial activity [27], [28]. However, such functional analyses are not reported till now for the cysteine rich PATE proteins.

The *Pate* gene cluster in the rat has received no attention. Among the eleven rat *Pate* gene sequences available in the GenBank, only Pate-B is reported, whereas the others are predicted. Further, no information is available about their expression pattern and functional significance. Though *Pate* genes are reported to be predominantly expressed in the testis and prostate, a recent study indicated their expression in the epididymis and not in the testis and prostate [22], suggesting a species specific expression pattern of these genes. Hence, it is very intriguing to determine the expression of rat *Pate* genes. In this study, we report the identification and characterization of ten rat *Pate* genes. Further, the expression profile of the *Pate* transcripts was analyzed and their androgen dependence determined. Since they are cysteine rich proteins and contain domains characteristic to venom proteins, their ability to kill bacteria was analyzed to demonstrate their possible contribution to the male reproductive tract immunity.

## Results

### *In silico* analyses

**Figure 1:**
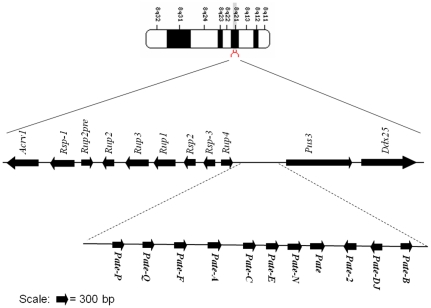
Genomic localization of rat *Pate* genes. Arrows indicate direction of transcription. Positions were taken from the Mapview (RGSC v3.4) at the National Center for Biotechnology Information (NCBI) website. Distance between genes is not to scale. Rat *Pate* sequences were submitted to GenBank and were assigned the accession numbers: *Pate-P* – JQ031758; *Pate-Q* – JF412807; *Pate-F* – JF412806; *Pate-A* – JF412804; *Pate-C* – HQ687475; *Pate-E* – JF412805; *Pate-N* – HQ687476; *Pate* – JF412809; *Pate-2* – HQ687477; *Pate-Dj* – HQ916281.

**Figure 2:**
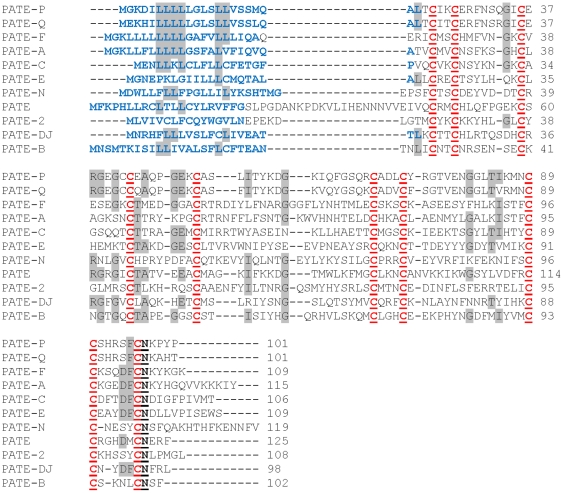
Multiple sequence alignment of PATE proteins. Alignment of rat PATE proteins. Signal peptide is shown in blue. The characteristic ten cysteines are indicated in red and underlined. The conserved amino acid residues are shaded.

Ten of the eleven (the exception being *Pate-B*, which is already reported in Gen Bank) rat *Pate* mRNA transcripts were amplified and sequenced. They are localized on chromosome 8q21 within a 2.5 kb segment present between the *Acrv1* and *Ddx25* genes, a characteristic feature observed in the humans and mice (Figure 1). PCR amplification using gene specific primers resulted in two amplicons each for *Pate* and *Pate*-2. Sequence analysis of the *Pate* amplicons revealed that the 378 bp amplicon corresponds to *Pate*, whereas the 400 bp amplicon seems to be its alternate transcript. Similarly, an alternate transcript for *Pate*-2 was also observed. The *Pate* sequences were submitted to GenBank and were assigned the accession numbers - *Pate-P* – JQ031758; *Pate-Q* – JF412807; *Pate-F* – JF412806; *Pate-A* – JF412804; *Pate-C* – HQ687475; *Pate-E* – JF412805; *Pate-N* – HQ687476; *Pate* – JF412809; *Pate-2* – HQ687477; *Pate-Dj* – HQ916281. Majority of them contained three exons (Figure S1). However, *Pate*, its alternate transcript, *Pate*-2 and its alternate transcript contained more than three exons. The number of exons reported in this study for each *Pate* transcript is in agreement with the information available in the rat genome. *In silico* protein translation analyses revealed that all the *Pate* mRNA transcripts except for the alternate transcripts of Pate and *Pate*-2, encode for proteins that are cysteine rich and contain the characteristic TFP/Ly-6/uPAR domain with a highly conserved distribution of 10 cysteines in two motifs (Figure 2). This is in agreement with the predictions available in the rat genome. Based on the ClustalW2 score, the homology among the rat PATE proteins was found to be high (Table 1). The rat PATE proteins are highly homologous to their known mouse and human counterparts (Table 2). All the *Pate* proteins identified in this study contain a signal peptide and seem to be secretory in nature (Figure S1). The predicted physical characteristics of the rat PATE proteins are given in Table 2. The alternate transcripts of *Pate* and *Pate*-2 contained a premature stop codon, because of which they do not encode the full length proteins (Figure S1) and hence were not characterized further.

**Table 1:** ClustalW2 score for rat PATE proteins.

	PATE-P	PATE-Q	PATE-F	PATE-A	PATE-C	PATE-E	PATE-N	PATE	PATE-2	PATE-Dj	PATE-B
**PATE-P**		82	28	26	25	24	16	21	17	27	22
**PATE-Q**			27	27	22	30	17	22	15	27	22
**PATE-F**				50	30	22	22	17	18	23	27
**PATE-A**					33	22	16	16	20	19	24
**PATE-C**						30	13	22	21	20	22
**PATE-E**							14	20	17	27	19
**PATE-N**								15	22	26	17
**PATE**									17	26	21
**PATE-2**										22	18
**PATE-DJ**											23
**PATE-B**											

**Table 2:** General characteristic features of rat PATE proteins.

Name of the protein	Length (aa)	MW (kD)	pI	Signal peptide	Net charge	O-Gly sites	Myristyltionsites	Phosphorylation			Homology with mouse counterpart	Homology with human counterpart
								Ser	Thr	Tyr		
PATE-P	101	11.04	8.31	1 to 21	−3	Nil	-Nil-	66	84	57	72%	–
PATE-Q	101	10.99	8.32	1 to 21	+3	Nil	-Nil-	66	84	57	71%	–
PATE-F	109	12.27	8.54	1 to 21	+4	Nil	-Nil-	79,83,86	Nil	58,87,106		–
PATE-A	115	12.98	9.27	1 to 23	+10	Nil	-Nil-	Nil	63	Nil	89%	–
PATE-C	106	11.9	6.68	1 to 19	0	Nil	-Nil-	27,72,80	68	28,82	73%	–
PATE-E	109	12.55	4.77	1 to 20	−6	Nil	-Nil-	Nil	27,51	29,68,80,82,95	28%	–
PATE-N	119	14.14	6.86	1 to 22	0	Nil	-Nil-	18,29,101	28,37,56	33,49,60,84,102	88%	–
PATE	125	14.35	9.07	1 to 21	+9	Nil	-Nil-	Nil	69	Nil	26%	62%
PATE-V1	99	11.3	8.53	1 to 21	+3	Nil	-Nil-	87,90	69	92	80%	62%
PATE-2	108	12.6	8.45	1 to 16	+4	Nil	-Nil-	66,74,99	45	58,70,101	83%	70%
PATE-DJ	98	11.56	9.08	1 to 18	+8	Nil	-Nil-	Nil	20,63	77	86%	63%
PATE-4	102	11.27	6.85	1 to 23	0	Nil	-Nil-	Nil	Nil	Nil	57%	38%

### *Pate* gene expression in the rat

**Figure 3:**
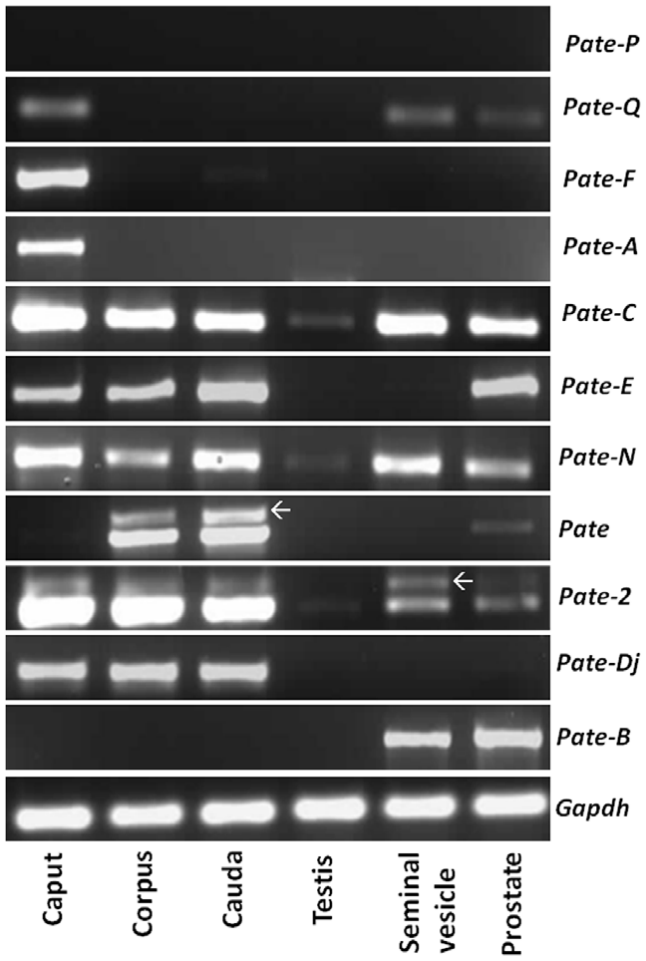
Expression of *Pate* genes in the male reproductive tract of rat. Total RNA isolated from the caput, corpus, cauda, testis, seminal vesicle and prostate of adult rats was reverse transcribed and *Pate* mRNAs amplified using gene specific primers. Arrows indicate the representative alternate transcripts of *Pate* and *Pate-2*.

**Figure 4:**
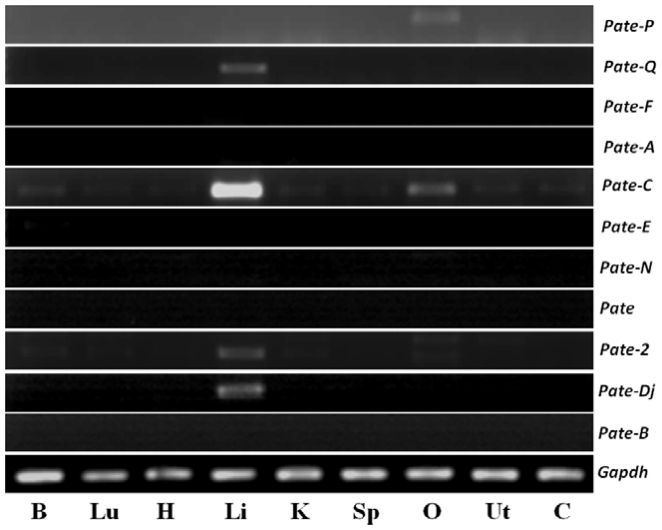
Tissue distribution of *Pate* genes in the rat. RT-PCR analysis was performed using total RNA isolated from **B**rain, **H**eart, **L**ung, **Li**ver, **K**idney, **Sp**leen, **O**vary, **Ut**erus and **Ce**rvix of adult rats.

Though *Pate* gene expression is thought to be prostate and testis specific, we found them to be expressed in the epididymis and seminal vesicle (Figure 3). *Pate-Q* mRNA was detected in the caput, seminal vesicle and prostate. Epididymis specific expression was observed for *Pate-F*, *Pate-Dj* and *Pate-A*. *Pate-C*, *Pate-N* and *Pate*-2 were found to be expressed in all the tissues analyzed. *Pate* and *Pate-E* expression was found to be present in the epididymis and prostate, whereas *Pate-B* was confined to seminal vesicle and prostate. *Pate-P* expression was not found in any of the male reproductive tract tissues analyzed (Figure 3). Since, the alternate transcripts of *Pate* and *Pate-2* are predicted to code for truncated proteins lacking the Ly-6 motif, we did not characterize them further. To determine if the expression pattern of *Pate* mRNA transcripts is male reproductive tract specific, RT-PCR was performed in a variety of tissues obtained from male and female rats (Figure 4). *Pate*, *Pate-F*, *Pate-A*, *Pate-E*, *Pate-B* and *Pate-N* were not found to be expressed in non-reproductive tissues of male rat and in the female reproductive tract tissues. However, *Pate-Q* and *Pate-Dj* expression was detected in the liver, whereas *Pate-P* was detected in the ovary. Weak expression of *Pate-2* and *Pate-C* was observed in majority of the tissues analyzed.

### Androgen dependent expression

**Figure 5:**
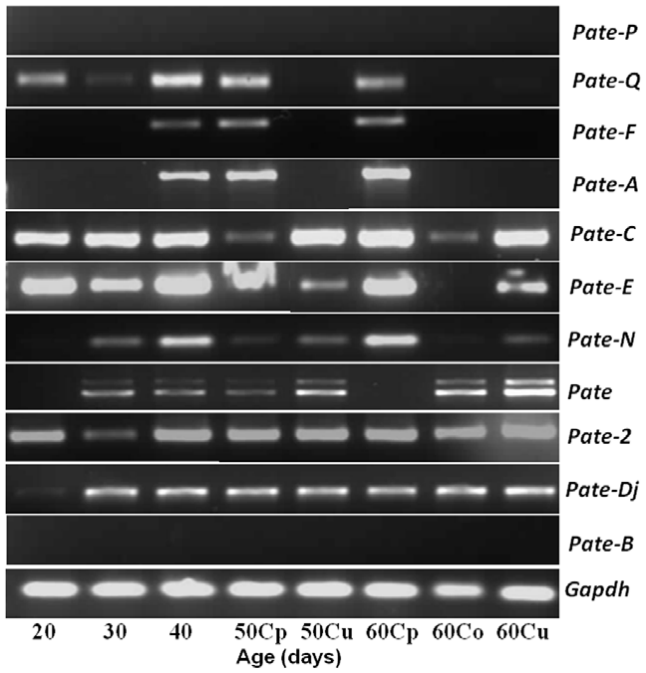
*Pate* gene expression in the epididymides of developing rats. RNA was isolated from the epididymides of 20–60 day old rats, reverse transcribed and PCR for *Pate* genes performed. Caput, corpus and cauda obtained from 50 and 60 day old animals are indicated as **Cp**, **Co** and **Cu** respectively.

Gene expression in the male reproductive tract is under the influence of androgens [29], [30]. To elucidate the effect of androgen variation, PCR analyses for *Pate*s were carried out using total RNA isolated from the epididymides of 20–60 day old rats. *Pate-Q*, *Pate-C*, *Pate-2*, *Pate-E* and *Pate-Dj* were found to be expressed in the epididymides throughout the development, where as *Pate-N* and *Pate* were expressed starting from 30 days (Figure 5). *Pate-F* and *Pate-A* expression in the epididymis seems to appear from 40 days during development. The absence of certain *Pate* transcripts in the caput or cauda during development (Figure 5) seems to correlate with the expression profile observed in the male reproductive tract tissues obtained from the 90 day old rats (Figure 3). For example, *Pate-Q* and *Pate-F* expression was restricted to the caput in the 90 day old rats and their expression is also not observed in the corpus and cauda during development. Similarly, *Pate* expression was absent in the caput of 90 day old rat and in the developmental regulation analyses, its expression is absent in the caput of 60 day old rat. These results indicate that expression of some of the *Pate* genes in the epididymis seems to be suppressed during development. *Pate-P* and *Pate-B* were not found to be expressed in the epididymides obtained from all the age groups, which is consistent with their absence in the epididymis of adult rat.

**Figure 6:**
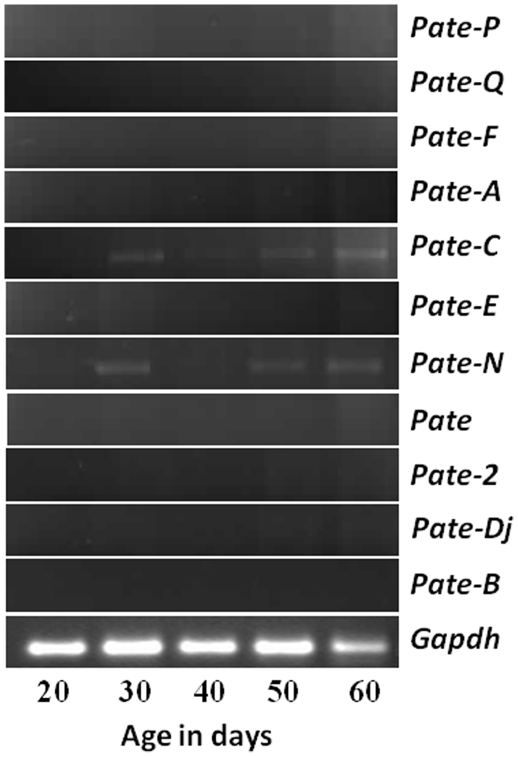
*Pate* gene expression in the testes of developing rats. RNA was isolated from the testes of 20–60 day old rats, reverse transcribed and PCR for *Pate* genes performed.

Though majority of the *Pate* genes were not expressed in the adult testis (Figure 3), it is possible that they may be expressed in younger rats during development. To determine, whether *Pate* genes have a role in testis development PCR analyses was carried out using mRNA isolated from testis of 20–60 day old rats. *Pate-C* and *Pate-N* were found to be weakly expressed starting from day 30 in the developing rats, whereas the other *Pate* genes were not detected at all the ages analyzed (Figure 6). The weak expression of *Pate-C* and *Pate-N* in the developing testes is consistent with the lower levels observed in the adult rats (Figure 3).

**Figure 7:**
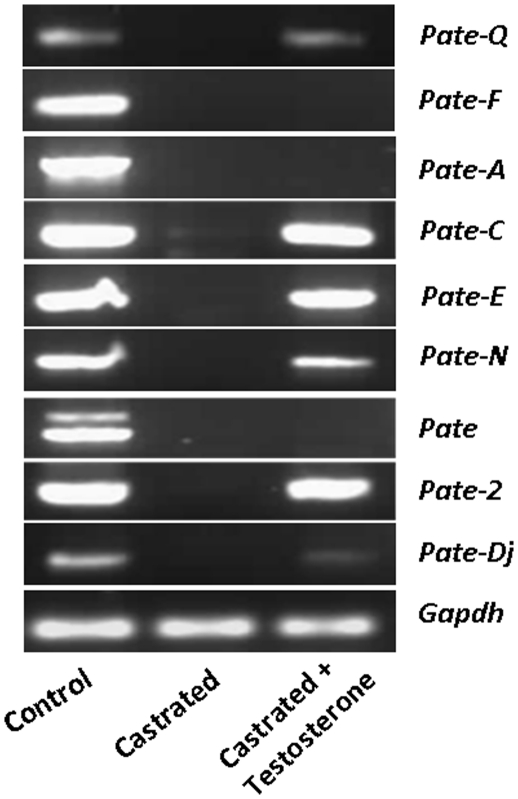
Androgen regulation of *Pate* gene expression in the adult rat. Adult male Wistar rats were either sham operated or castrated or castrated and DHT supplemented. Two weeks after castration total RNA was isolated from the epididymides and PCR analyses carried out for the expression of *Pate* genes.

To gain further knowledge into the role of androgens in regulating *Pate* gene expression, RT-PCR analyses was performed using epididymides obtained from androgen ablated rats with or without testosterone supplementation. PCR analyses were performed for those *Pate* genes that were expressed in the adult epididymis. Androgen ablation resulted in complete loss of *Pate* gene expression (Figure 7). Dihydrotestosterone supplementation reverted the expression of majority of the *Pate* genes except *Pate*, *Pate-A* and *Pate-F*. These results suggest that *Pate* genes are under the control of androgens in the epididymis.

### Three-dimensional structure prediction

**Figure 8:**
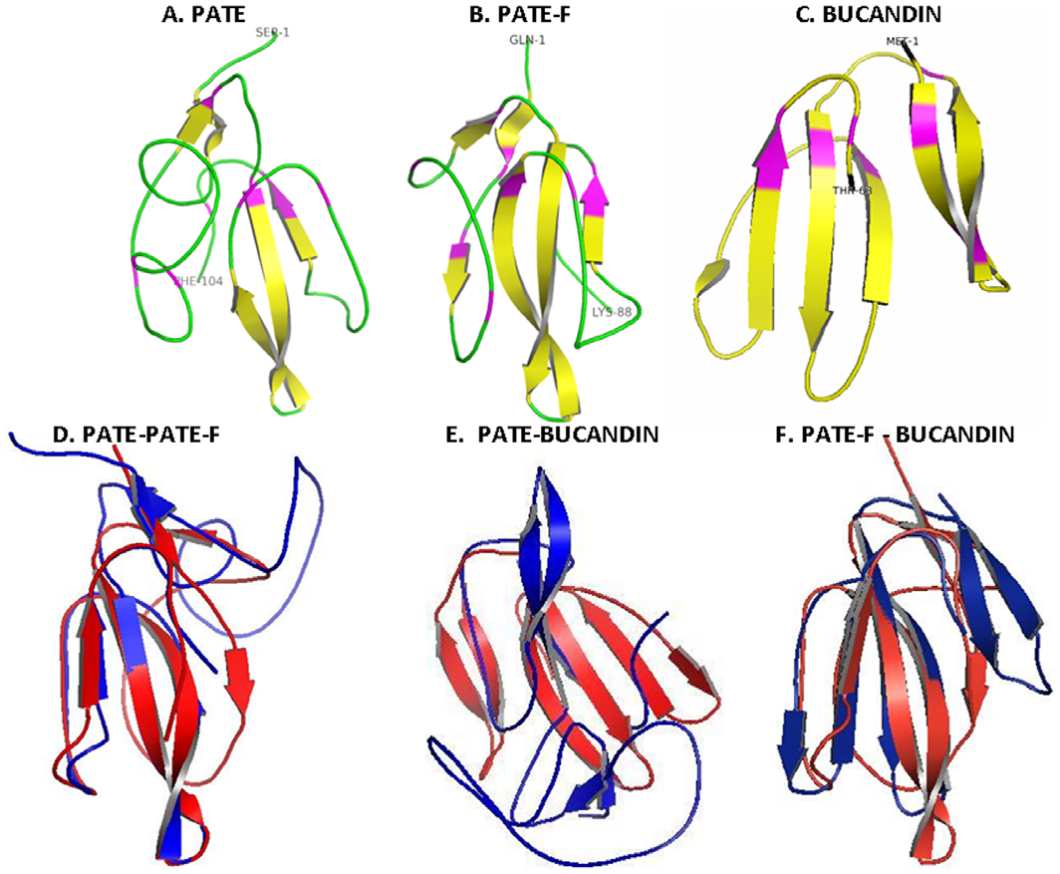
Three dimensional protein structure predictions. Protein sequences were submitted to FUGUE threading server and the best model was selected based on best Z score value obtained. The final structure of PATE and PATE-F was generated using the best model by modeler 9.10. Purple regions indicate conserved cysteines. Cartoon models of **A**) rat PATE, **B**) rat PATE-F, **C**) Bucandin, **D**) PATE (blue) and PATE-F (red) superimposed, **E**) PATE (blue) and Bucandin (red) superimposed and **F**) PATE-F (red) and Bucandin (blue) superimposed.

Among the *Pate* genes identified, we chose *Pate* and *Pate-F* because of their predominant expression in the epididymis for further characterization. Three-dimensional (3D) modeling of mature PATE and PATE-F proteins displayed structure similar to the three fingered toxin, bucandin (Figure 8A–C), a neurotoxin. PATE and PATE-F in different orientations are shown (Figure S2). Superimposition of the 3D structures of PATE and PATE-F reveal that they match to a larger extent (Figure 8D). PATE-F structure is in agreement with bucandin structure (Figure 8F). PATE structure also seems to be matching with bucandin structure (Figure 8E), but not to an extent to that of PATE-F, because of the additional amino acid sequence coded by the extra exon. The root mean square distance (RMSD) value for PATE and PATE-F superimposition was 1.93, suggesting structural similarities between these proteins. However, the RMSD values for PATE-Bucandin and PATE-f-Bucancin superimpositions were 3.5 and 2.9 respectively. Basing on the manual alignment of PATE, PATE-F and Bucandin, it can be expected that five disulfide bridges could be formed similar to that in Bucandin (Figure S3). Ramachandran plot analyses revealed that the percentage of amino acids in the allowed regions for PATE and PATE-F was 78.9 and 79.5 respectively (data not shown). There were no amino acids (0 percent) in the disallowed regions. The G factor values for PATE and PATE-F were −0.57 and −0.29 respectively. All the three proteins contain three fingered structure. ClustalW2 analysis revealed that the similarity score between bucandin and PATE was 22 and it was 14 between bucandin and PATE-F. Though there is less of sequence similarity among these proteins, they seem to have conserved the organization of 10 cysteine residues that may contribute to their structural similarity.

### Immunolocalization

**Figure 9:**
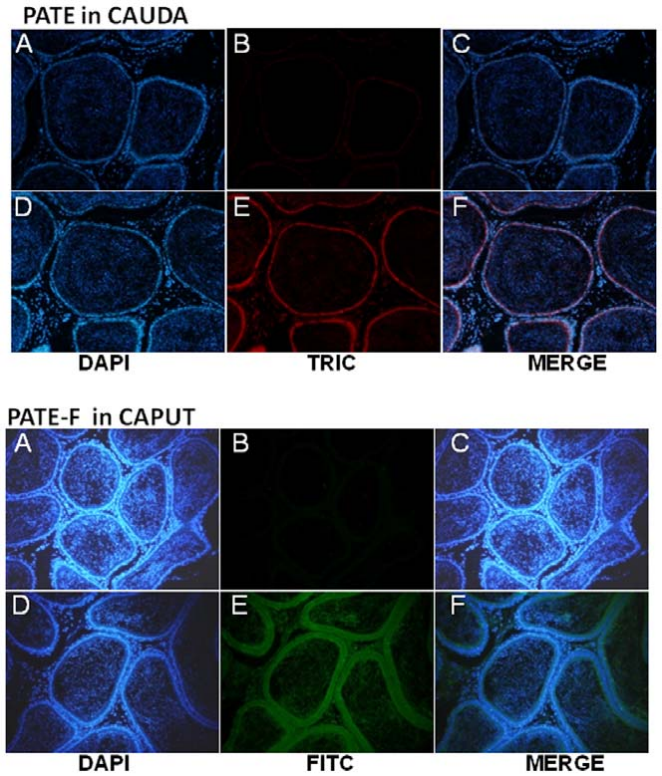
Immunolocalization of rat PATE and PATE-F in the epididymis. Serial sections of the rat tissues were subjected to antigen retrieval in citrate buffer pH 6.0. They were then probed with polyclonal antibodies (1∶250 dilution) raised in rabbit against PATE and PATE-F followed by TRIC (for PATE) or FITC (for PATE-F) conjugated secondary antibody (1∶500 dilution) against rabbit IgG raised in goat. Sections were counter-stained with DAPI. Panels A–C – preimmune serum; D–F – immune serum. Magnification – 10×.

To allow further insight into the functional role of PATE proteins in male reproductive tract and to confirm whether their mRNA are translated into proteins, we analyzed their expression using immunofluorescence techniques. PATE protein was found to be abundantly localized in the cauda (Figure 9) and appears to be present throughout the epithelium, suggesting that it may be secreted into the lumen. PATE-F protein was also found to be expressed abundantly in the caput (Figure 9). Similar to PATE, PATE-F is localized throughout the epithelium and on the spermatozoa.

**Figure 10:**
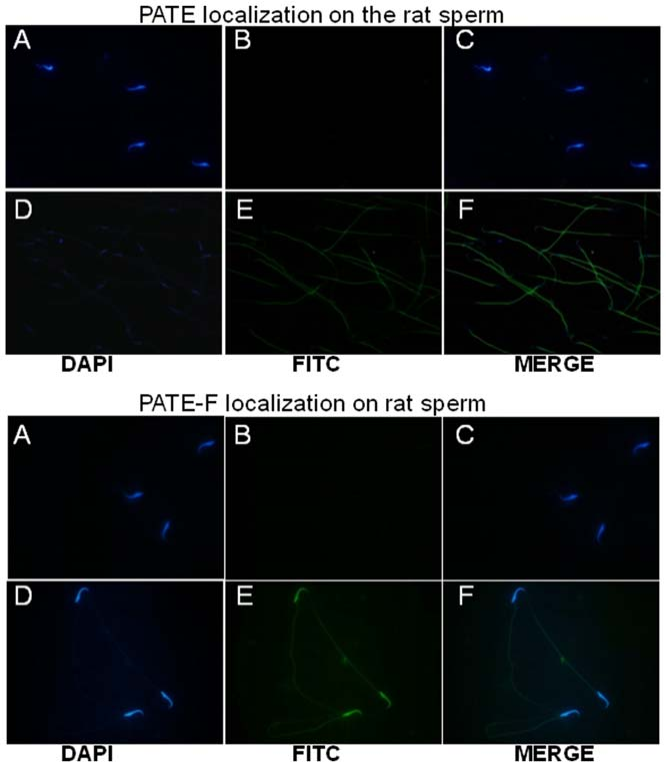
Immunofluorescence detection of PATE and PATE-F on rat sperm. Cauda epididymides from adult rats were dissected out and the spermatozoa collected were air dried and fixed on glass slides by methanol. PATE and PATE-F localization was carried out by incubating with PATE and PATE-F polyclonal antibodies raised in rabbit followed by FITC conjugated secondary antibodies against rabbit IgG raised in goat. Counter staining was carried out using DAPI. **A–C**, preimmune serum. **D–F**, immune serum. Magnification – 60×.

Localization of PATE and PATE-F proteins on the sperm was analyzed using immunofluorescence. PATE was found to be present throughout the sperm surface, whereas, PATE-F localization was restricted to the head region (Figure 10). Presence of PATE proteins on the sperm surface indicates their possible role in spermatogenesis and sperm maturation.

### Antimicrobial activity

**Figure 11:**
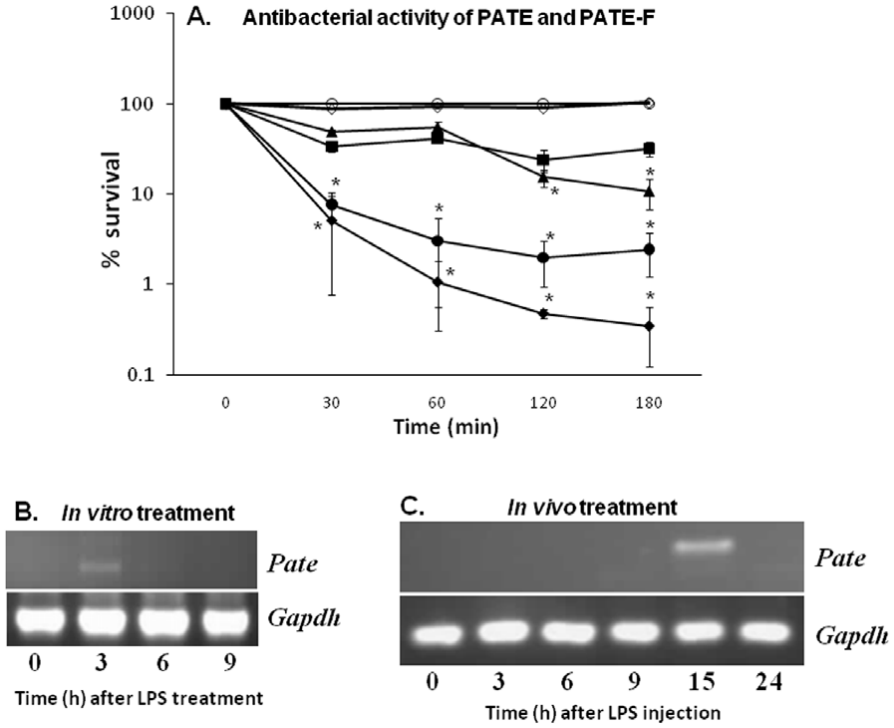
Antibacterial activities of rat PATE and PATE-F and their response to endotoxin challenge. **A,** Mid-log phase *E. coli* were incubated with 0 (○), 10 (▪), 25 (▴), 50 (•) and 100 (♦) µg/ml rat recombinant PATE or 100 µg/ml of PATE-F (◊) protein for 0–180 min. After incubation, the assay mixture was serially diluted with 10 mM phosphate buffered saline (pH 7.4), plated on LB plates and incubated overnight. Colonies were hand counted. Values shown are Mean ± S.D. * indicates p<0.05. **B,** Rat epididymides were maintained in nutritive medium (136.89 mM NaCl, 5.63 mM KCl, 1.80 mM CaCl_2_, 0.36 mM NaH_2_PO_4_, 14.88 mM NaHCO_3_ and 5.55 mM glucose pH 7.6–7.8) and challenged with LPS (1 µg/ml) for 0, 3, 6 and 9 h. RNA was isolated after LPS treatment and the expression of rat *Pate* in the epididymides was analyzed by RT-PCR. **C,** Rats were challenged with a single intraperitoneal dose (1 mg/kg body weight) of LPS and epididymides were collected at 0, 3, 6, 9, 15 and 24 h after injection. RNA isolated from the epididymides was used for *Pate* gene expression.

Proteins with cationic nature or rich in cysteine content like eppin and WFDC proteins are known to exhibit antimicrobial activity [27], [28]. Because of their cationic nature (pI = 9.0 for PATE and pI = 8.54 for PATE-F) and high cysteine content they might be expected to exhibit antimicrobial activity. PATE at higher concentrations (50 and 100 µg/ml) exhibited bacterial killing activity, whereas PATE-F even at a concentration of 100 µg/ml did not display any antibacterial activity (Figure 11A).

### Endotoxin effect on *Pate* expression

Since the recombinant PATE protein showed potent antibacterial activity *in vitro*, its possible functional role during infection or under conditions that mimic an infection to counter pathogen attack in the male reproductive tract was investigated. To accomplish this, *Pate* expression was analyzed by semi-quantitative RT-PCR in the cauda epididymides challenged with LPS *in vitro*. Though *Pate* expression is not observed in the untreated control, it was up regulated 3 hours after LPS challenge followed by a decline at the later time points (Figure 11B). To determine whether, the effects observed *in vitro* are replicated *in vivo*, *Pate* expression was analyzed in the cauda epididymides obtained from rats challenged with a single dose of LPS. *Pate* mRNA levels were found to be increased 15 h after LPS injection (Figure 11C). These results suggest that *Pate* may have a role in contributing to the innate immune responses of the male reproductive tract during endotoxin challenge.

## Discussion

Epididymal fluid is a complex mixture of proteins [31] that is thought to modify sperm surface during epididymal transit and maturation. PATE proteins, though reported to be present in the male reproductive tract of humans and mouse, their role in this organ system still remains elusive. The expression pattern of *Pate* genes and proteins are not characterized in the rat. In this study, we analyzed their mRNA and protein expression to determine their possible roles in male reproductive tract function and immunity.

*In silico* analyses revealed that all the ten *Pate*s identified in ths study are clustered on chromosome 8 within a 2.5 kb segment present between the *Acrv1* and *Ddx25* genes. Similar organization of *Pate* genes is observed in the mouse and humans [21], [22]. Further, majority of the *Pate* mRNAs are encoded by three exons, whereas *Pate* and *Pate*-2 are coded by five and four exons respectively. This is in agreement with the gene structure available at GenBank. Similar exon structure was reported in the human and mouse, wherein *Pate* and *Pate-2* were encoded by five and four exons respectively and the rest of the *Pate*s are encoded by three exons, adding further evidence that these gene are highly conserved among the species. Sequence analyses of the alternate transcripts of rat *Pate* and *Pate-2* revealed that they encode for truncated proteins lacking the ten cysteine signature and. could have functional roles that are different than their counterparts.

A high degree of homology among the rat PATE proteins suggests a common physiological function, such as regulating the activities of ion-channels, as was demonstrated for the human PATE-B, mouse PATE-C and PATE-B [21], [26]. Rat PATE proteins can be classified to the secreted Ly-6 family, because of their 10 cysteine signature and the presence of three fingered protein structure. The similarity of PATE and PATE-F three dimensional structures to the toxin bucandin a 63 amino acid neurotoxin isolated from the Malaysian krait (*Bungarus candidus*) [32] gives a clue to understand the role of PATE and PATE-F in nerve function. The RMSD value (1.9) obtained when PATE and PATE-F 3D structures were superimposed is a clear indication that these proteins share structural simililarity. PATE-Bucandin and PATE-F-Bucandin protein superimpositions resulted in RMSD values of 3.5 and 2.9 respectively; values that do not indicate a high degree of similarity between PATE proteins and Bucandin. In our analyses, we did not apply in depth the various factors (compactness, hydrogen bonding, percent secondary structure, principal component analyses, etc) that affect the RMSD and this could have resulted in high values.

To the best of our knowledge, we report for the first time the expression pattern of *Pate* mRNA transcripts in the rat. *Pate* gene expression is not restricted only to the prostate and testis, as indicated by their nomenclature. However, organ (epididymis) specific expression of rat *Pate* (*Pate-F* and *Pate-A*) was observed in our study similar to the mouse, wherein *Pate-F* and *Pate-Dj* were testis specific and *Pate-Q* and *Pate-P* were restricted to the placenta [21]. On the same lines, the human *Pate-Dj* mRNA was detected only in the testis. Reproductive tract specific expression was also demonstrated for other genes such as *Spag11e* [33], *DEFB118* [34] and members of the HE2 family [10]. The expression of rat *Pate* genes in non-reproductive and female reproductive tissues suggests that they may have functions beyond male reproductive tract physiology. Such observations were reported for the mouse and human *Pate* genes [21], [22].

Hormonal regulation of a wide variety of genes due to fluctuations of androgens at various stages of development in the male reproductive system is well reported [35]. Androgen levels in the epididymis of rat decline from birth until 20 days and a normal level of 10 ng/g tissue (35 nM) is maintained until approximately 40 days after which, the levels begin to increase to that of the adult, between 15–20 ng/g [36]. Serum testosterone levels in the young rat remain low and do not begin to increase to adult levels until 35–40 days of age [37]. Absence of *Pate-A*, *Pate-F*, *Pate-N* and *Pate-Dj* in the epididymides during early development (20–30 days) correlate with reported low levels of androgens. *Pate-Q*, *Pate-F* and *Pate-A* in the adult rat (90 days old) was restricted to the caput. In the developing rat epididymis also, their expression was restricted to the caput in the 50 and 60 day old rats. The expression observed in the 30 and 40 day old animals could be due to the use of whole epididymides . It is possible that the expression of these genes could be restricted to the caput in the younger animals also. Presence of some of the *Pate* mRNA transcripts at all the stages of development indicated their androgen independent expression in the epididymis. Variation in testicular androgens during development in the rat is quite different from the epididymis. A steady increase in testosterone levels occurs in the rete testis of 30–130 day old rats [38], [39]. Though majority of the *Pate* mRNA were not detected in the adult rat, we analyzed their expression in the testis to determine whether they are expressed in the developing rats and whether they have a possible role in testicular development. Among the *Pate* genes (*Pate-N*, *Pate-C* and *Pate-2*), whose expression was barely detected in the adult rats, *Pate*-C and *Pate*-N were found to be expressed at very low levels in the testes of developing rats starting from day 30. The expression pattern of *Pate* transcripts in the testis is androgen dependent since it correlates with the minimal androgen levels from day 20 to day 40 and increased androgen in the adult [36].

The role of androgens in governing *Pate* gene expression was evident since a down regulation was observed in the epididymides of castrated rats and that DHT supplementation reverted the mRNA levels. *Pate* gene expression in relation to androgens was reported in the human and mouse. In the human dorsal prostate, PATE-B and PATE-E were found to be up regulated in castrated rats, whereas in the ventral prostate, no changes were observed for PATE-H under the same conditions [21]. In the mouse, mixed responses in *Pate* gene expression was observed in the initial segment, caput and proximal epididymis of gonadectomized mice [22]. Androgen regulation of *Pate* genes seem to vary among the species and in the organs within species, suggesting a more complex network of regulatory mechanisms that may include the testicular factors.

PATE and PATE-F proteins were found to be abundantly localized in the male reproductive tract and on the spermatozoa. Similar PATE protein expression in the reproductive tract and on the sperm is reported in the mice and humans [19], [21], [22] implicating that they may have similar functions. Further, the exact role of PATE proteins in the male reproductive tract remains elusive, though their possible role in calcium transport to regulate acrosome reaction is reported [26]. In this study, PATE was found to be predominantly localized on the sperm tail, whereas PATE-F was restricted to the sperm head. Human PATE and PATE-B were found to be localized only on the sperm head [20], [21]. The presence of PATE-F specifically on the sperm head suggests that it may be involved in fertilization, whereas PATE localization on the tail sperm surface may contributes to motility.

Cysteine rich proteins belonging to the WFDC family, eppin and defensins are known to exhibit potent antimicrobial activity and the mechanisms involve permeabilization of bacterial membranes and inhibition of macromolecular synthesis [27], [28]. Further, snake toxins that contain the Ly-6 domain are shown to be involved in defense against microbes [40]. In this study, we report that rat PATE exhibited potent antimicrobial activity, a property that is highly conserved in LY-6 family of proteins; and PATE may function to confer antimicrobial defense mechanisms in the male reproductive tract. PATE-F, on the other hand, did not exhibit any antimicrobial activity, suggesting a varied functional nature among the PATE proteins.

Since PATE protein displayed potent antimicrobial activity, its epididymal expression was analyzed in response to LPS *in vitro* and *in vivo*. We observed an induction of *Pate* gene expression during LPS challenge, suggesting that the innate immune responses in the male reproductive tract under these conditions may involve alterations in *Pate* mRNA expression. Ours is the first study that provides evidence on the possible involvement of *Pate* genes in the male reproductive tract immunity. However, the expression profile of PATE protein in response to LPS challenge or bacterial infection and its ability to clear the invading pathogens needs further investigation.

Based on the results of this study, we conclude that *Pate* genes are abundantly expressed in the male reproductive tract and are androgen dependent. Their presence on the sperm and their ability to be antimicrobial and respond to endotoxin challenge implicates a role in fertility and male reproductive tract defense mechanisms as well.

## Materials and Methods

### *In silico* analyses

The rat *Pate* predicted sequences were obtained from the rat genome (build RGSC v3.4) at the NCBI website (http://www.ncbi.nlm.nih.gov/). HUGO nomenclature was followed for the gene and protein notation used in this study. Gene symbols are italicized, with only the first letter in uppercase and the remaining letters in lowercase (*Pate*). Protein designations are the same as the gene symbol; all uppercase, but are not italicized (PATE). Gene specific primers were designed for each *Pate* mRNA (Table 3). RT-PCR was performed using rat testis and epididymis mRNA as the template. The *Pate* PCR amplicons were sequenced, aligned and deposited in GenBank. The corresponding exon/intron boundaries were determined by aligning the cDNA with the genomic sequence. The sequences were translated and the predicted physical features of the deduced amino acid sequences were analyzed using tools available at ExPASy proteomics server (http://ca.expasy.org/).

**Table 3:** Gene specific primers used in this study.

Gene Name	Forward primer(5′ → 3′)	Reverse Primer(5′ → 3′)
*Pate-P*	**ATGGGAAAGGACATCTTGCTGCTC**	**TCACGGATATGGTTTATTGCAG**
*Pate-Q*	**ATGGAAAAGCACATCTTGCTGCTCC**	**TCAGGTATGTGCTTTATTGCAG**
*Pate-F*	**AGACTGAGATGGGCAAGCT**	**ACCTCTTGCATTGAAAAGATAG**
*Pate-A*	**GAGTCTGCAACCTGCTCTCATCTA**	**ATGTTGGGGTACAGCCAGA**
*Pate-C*	**ATGGAAAACCTACTGAAGCTGTGCC**	**CTAAGTCATTACGATTGGAAAACCG**
*Pate-E*	**ACCTGCTGCAAGAATTAGAAGATCC**	**AAGATGAGGAAGACTGGAGG**
*Pate-N*	**ATGGACTGGCTCCTGTTTCTTTTG**	**TTAGACAAAATTATTTTCTTTGAAGTG**
*Pate*	**ATGTTCAAGCCCCACTTACTGAG**	**CTAGAAAAAGAATGCTTTCCCTGTGAC**
*Pate-2*	**ATGCTGGTGATAGTCTGTTTGTTCTGCC**	**CTAGAGTCCCATTGGGAGGTT**
*Pate-Dj*	**ATGAACAGGCACTTCTTGCTGCTC**	**TTAAAGTCTGAAGTTACAAAAATC**
*Pate-B*	**CAAGCCACCTTCTAACATCCA**	**GCTTAAAACAACACGCAT**

To generate the possible three dimensional structures of PATE and PATE-F, their protein sequences were initially submitted for BLAST against PDB database to search for homologous proteins. In the absence of any homologous proteins to PATE and PATE-F in the existing database even with the identity of TWILIGHT region, the sequences were submitted to FUGUE threading server. The parameters are set to generate the five best models and the best model is selected based on best Z score value obtained. This model was taken as the template to generate the final structure of PATE and PATE-F by using modeler 9.10. There were 100 models with different energy constituents generated, of which we selected the models with lowest energy constituents. In order to refine these residues ModLoop database was used to address the amino acid residues that fall in the disallowed regions based on the Ramachandran plot values. The obtained structure was energy minimized in the Gromos Force field by Gromacs software.

### Tissue specimens and RT-PCR

All the animals used in the study were obtained from National Institute of Nutrition, Hyderabad, India. Tissues collected from Wistar rats (aged 20–90 days; n = 3) were placed in RNA*Later* (Ambion Inc, Austin, TX, USA) solution overnight at 4°C to allow penetration and fixation and stored at −70°C. Total RNA was extracted using the TRIzol reagent (Invitrogen, Carlsbad, CA, USA) from the following tissues: the three regions of the epididymis (caput, corpus and cauda), testis, prostate, seminal vesicle, brain, liver, lung, kidney, heart, spleen, cervix, ovary and uterus. Total RNA (2 µg) was reverse transcribed using 200 U SuperSciptIII (Invitrogen, Carlsbad, CA, USA) and 0.5 µg of oligodT (Invitrogen, Carlsbad, CA, USA) according to the manufacturer's instructions. 2 µl of the resultant cDNA was amplified by PCR using gene specific primers (Table 3). A typical PCR reaction consisted of the following conditions: 94 C for 2 min followed by 25–35 cycles at 94 C for 30 sec, 56 C for 30 sec and 72 C for 30 sec, and with a final round of extension at 72 C for 10 min. PCR amplicons were analyzed on 2% agarose gels and sequenced. To study the expression of *Pate* transcripts under androgen ablated conditions, epididymides were obtained from sham operated, castrated and castrated + DHT supplemented Wistar rats (n = 5 in each group). DHT pellet (20 mg) was implanted subcutaneously after castration to supplement testosterone. All the animals were sacrificed 14 days after castration.

### Recombinant protein production

Recombinant PATE and PATE-F proteins were prepared as described earlier [41]. DNA corresponding to the open reading frame of PATE and PATE-F full length without the signal peptide were cloned into pQE30 expression vector (Qiagen, Valencia, CA, USA). *E. coli* (BL-21) was transformed with pQE30 vector containing rat *Pate or Pate-F* cDNA according to the supplier's instructions. His tagged protein expression was induced with 1 mM isopropyl-1-thio-β-D-galactoside for 3 h at 37°C. 1% glucose was maintained in the medium to avoid baseline expression of the protein prior to induction. Bacterial cells were lysed with buffer A (100 mM NAH_2_PO_4_, 10 mM Tris-Cl, 6 M gunadium hydrochloride, pH 8.0). Bacterial lysate was then incubated with nickel-nitrilotriacetic acid-agarose (Qiagen) for 1 h to allow binding of His-tagged recombinant protein to the resin. It was then transferred to a column, washed with buffer B (100 mM NAH_2_PO_4_, 10 mM Tris-Cl, 8 M urea, pH 8.0 and the recombinant protein eluted with buffer B of varying pHs namely 6.3, 5.9 and 4.5. The His-tagged recombinant PATE proteins contained the following additional amino acid residues at the N-terminus (MRGSHHHHHHGS) due to the construction of the vector. Fractions were analyzed on 15% gradient polyacrylamide Tris-Tricine gels and stained with Coomassie blue G250. Further, the identity of the protein was confirmed by Western blotting using anti-His-tag antibody. To remove urea, fractions containing purified protein were pooled and dialyzed serially in 10 mM phosphate buffered saline (pH 7.4) containing decreasing concentration of urea to allow protein refolding.

### Antibody production and immunodetection

Rat PATE and PATE-F antibodies were raised in our laboratory. Briefly, recombinant PATE or PATE-F protein was mixed with complete adjuvant and rabbits were immunized followed by booster doses 4 and 6 weeks after initial immunization. Antiserum was collected 2 weeks after the second booster dose. The specificity of antiserum obtained was confirmed by Western blotting using positive and negative controls. As a positive control, recombinant protein (PATE or PATE-F) was used. Anti serum to PATE or PATE-F recognized their respective recombinant proteins (Figure S4). In negative control experiments, antiserum to PATE was tested against PATE-F and vice-versa. No cross reactivity was observed (Figure S4).

For immuno fluorescent staining, epididymides and testes were fixed in 4% paraformaldehyde and Bouin's fluid respectively and embedded in paraffin. Five micron thick sections were taken and treated with xylene and graded alcohol (70–100%). The sections were subjected to antigen retrieval in citrate buffer pH 6.0. PATE and PATE-F were detected by incubating the sections using polyclonal antibodies (1∶250 dilution) raised in rabbit followed by TRIC (for PATE) or FITC (for PATE-F) conjugated secondary antibody (1∶500 dilution) against rabbit IgG raised in goat. Sections were counter-stained with DAPI. For immunostaining of the sperm, adult rat epididymides were dissected out and the spermatozoa were air dried and fixed on glass sides using methanol. Immunofluorescence on the sperm was detected by using PATE or PATE-F antiserum and anti-rabbit secondary antibodies tagged with FITC. Counter staining was done using DAPI. Photographs were taken using a color digital imaging system attached to a Leica Photomicroscope. The magnifications were 10 and 60× for tissues and sperm respectively. Surgical procedures were conducted using the guidelines for the care and use of laboratory animals and this study was specifically approved by the Institutional Animal Ethics Committee of University of Hyderabad.

### Antibacterial assay

Colony forming units (CFU) assay was employed to test the antibacterial activity as described previously [41]. *E. coli* XL-1 Blue (Stratagene, La Jolla, CA, USA) grown to mid-log phase (*A*_600_ = 0.4–0.5) diluted with 10 mM sodium phosphate buffer (pH 7.4) was used in the assay. Varying concentration of PATE or PATE-F protein (10–100 µg/ml) was added to approximately 2×10^6^ CFU/ml of bacteria and incubated at 37°C for 30–120 min. After incubation, the assay mixtures were serially diluted with 10 mM sodium phosphate buffer (pH 7.4) and 100 µl of each was spread on a Luria–Bertani agar plate and incubated at 37°C overnight to allow full colony development. The resulting colonies were hand counted and plotted as % survival. Values shown are Mean ± S.D. Statistical analyses were performed using Student's t-test available in Sigma Plot software.

### In vitro and in vivo endotoxin treatments

The effect of endotoxin challenge on *Pate* expression was investigated *in vitro* and *in vivo* following the methodology described earlier [42], [43]. For the *in vitro* experiments, caput, cauda and testis from adult rats were dissected and cut into two longitudinal halves. One half of the tissue was used as control, and the other was treated with LPS. Tissues were transferred to 2 ml nutritive media (136.89 mM NaCl, 5.63 mM KCl, 1.80 mM CaCl_2_, 0.36 mM NaH_2_PO_4_, 14.88 mM NaHCO_3_ and 5.55 mM glucose pH 7.6–7.8) and cultured at 30 C with aeration. After 15 min of incubation, tissues were transferred to nutritive solution with or without LPS (1 µg/ml) and incubated for 0–9 h. During these incubations nutritive solution with or without LPS were renewed every 30 min. The tissues were collected, rinsed with PBS, frozen in liquid nitrogen and stored in −80 C until use.

For the *in vivo* LPS challenge, adult male Wistar rats (90-days-old), maintained on a 12L∶12D lighting schedule, at 22–25°C, with food and water ad libitum, were injected intraperitoneally with LPS (1 mg/kg body weight; from *E. coli* 0111∶B4; Sigma, St. Louis, MO, USA) or saline (control). LPS dose and site of injection was chosen based on previous reports [43]. Rats were sacrificed at 3, 6, 9, 15 and 24 h after LPS treatment. Cauda epididymis was identified, stripped of connective tissues, frozen in liquid nitrogen, and kept at −70°C until use.

## Supporting Information

Figure S1 **Alignment of rat** ***Pate*** **mRNA and chromosomal sequence.** Bold upper case letters indicate exons and lower case letters indicate introns. Amino acids are indicated in bold single letters. Predicted signal peptide cleavage site is underlined and in italics. The conserved ten cysteines are indicated in blue and underlined. Numbers in parentheses indicate amino acids of the protein.
(DOC)

Figure S2 **Three dimensional structures of rat PATE and PATE-F.** The ten conserved cysteines are shown. Pairs of cysteines that may take part in disulfide bonding are shown in same color.
(PPT)

Figure S3 **Manual alignment of PATE, PATE-F and Bucandin.** Cysteines in same color may form disulphide bonds in PATE and PATE-F similar to that of Bucandin.
(DOC)

Figure S4 **Western blotting using positive and negative controls.** PATE and PATE-F recombinant proteins were probed with the polyclonal antibodies. For PATE, PATE-F was used as a negative control and vice-versa.
(TIF)

## References

[1] Ducheux J, Gatti J, Dacheux F (2003). Contribution of epididymal secretory proteins for spermatozoa maturation.. Microscopy Research and Technique.

[2] Peitz B (1988). Effects of seminal vesicle fluid components on sperm motility in the house mouse.. Journal of Reproduction and Fertility.

[3] Peitz B, Olds-Clarke P (1986). Effects of seminal vesicle removal on fertility and uterine sperm motility in the house mouse.. Biology of Reproduction.

[4] Robert M, Gagnon C (1996). Purification and characterization of the active precursor of a human sperm motility inhibitor secreted by the seminal vesicles: identity with semenogelin.. Biology of Reproduction.

[5] Hall SH, Hamil KG, French FS (2002). Host defense proteins of the male reproductive tract.. J Androl.

[6] Yenugu S, Hamil KG, Radhakrishnan Y, French FS, Hall SH (2004). The androgen-regulated epididymal sperm-binding protein, human beta-defensin 118 (DEFB118) (formerly ESC42), is an antimicrobial beta-defensin.. Endocrinology.

[7] Yenugu S, Chintalgattu V, Wingard CJ, Radhakrishnan Y, French FS (2006). Identification, cloning and functional characterization of novel beta-defensins in the rat (Rattus norvegicus).. Reprod Biol Endocrinol.

[8] Hamil KG, Liu Q, Sivashanmugam P, Anbalagan M, Yenugu S (2003). LCN6, a novel human epididymal lipocalin.. Reprod Biol Endocrinol.

[9] Travis SM, Anderson NN, Forsyth WR, Espiritu C, Conway BD (2000). Bactericidal activity of mammalian cathelicidin-derived peptides.. Infect Immun.

[10] Hamil KG, Sivashanmugam P, Richardson RT, Grossman G, Ruben SM (2000). HE2beta and HE2gamma, new members of an epididymis-specific family of androgen-regulated proteins in the human.. Endocrinology.

[11] Hamil KG, Liu Q, Sivashanmugam P, Yenugu S, Soundararajan R (2002). Cystatin 11: a new member of the cystatin type 2 family.. Endocrinology.

[12] Blankenvoorde MF, van't Hof W, Walgreen-Weterings E, van Steenbergen TJ, Brand HS (1998). Cystatin and cystatin-derived peptides have antibacterial activity against the pathogen *Porphyromonas gingivalis*.. Biol Chem.

[13] Hiemstra PS, Maassen RJ, Stolk J, Heinzel-Wieland R, Steffens GJ (1996). Antibacterial activity of antileukoprotease.. Infect Immun.

[14] Collard MW, Griswold MD (1987). Biosynthesis and molecular cloning of sulfated glycoprotein 2 secreted by rat Sertoli cells.. Biochemistry.

[15] Griswold MD, Roberts K, Bishop P (1986). Purification and characterization of a sulfated glycoprotein secreted by Sertoli cells.. Biochemistry.

[16] Zhang K, Gao R, Zhang H, Cai X, Shen C (2005). Molecular cloning and characterization of three novel lysozyme-like genes, predominantly expressed in the male reproductive system of humans, belonging to the c-type lysozyme/alpha-lactalbumin family.. Biology of Reproduction.

[17] Mandal A, Klotz KL, Shetty J, Jayes FL, Wolkowicz MJ (2003). SLLP1, a unique, intra-acrosomal, non-bacteriolytic, c lysozyme-like protein of human spermatozoa.. Biology of Reproduction.

[18] Jalkanen J, Huhtaniemi I, Poutanen M (2005). Mouse cysteine-rich secretory protein 4 (CRISP4): a member of the Crisp family exclusively expressed in the epididymis in an androgen-dependent manner.. Biology of Reproduction.

[19] Bera TK, Maitra R, Iavarone C, Salvatore G, Kumar V (2002). PATE, a gene expressed in prostate cancer, normal prostate, and testis, identified by a functional genomic approach.. Proceedings of the National Academy of Sciences of the United States of America.

[20] Soler-Garcia AA, Maitra R, Kumar V, Ise T, Nagata S (2005). The PATE gene is expressed in the accessory tissues of the human male genital tract and encodes a secreted sperm-associated protein.. Reproduction.

[21] Levitin F, Weiss M, Hahn Y, Stern O, Papke RL (2008). PATE gene clusters code for multiple, secreted TFP/Ly-6/uPAR proteins that are expressed in reproductive and neuron-rich tissues and possess neuromodulatory activity.. Journal of Biological Chemistry.

[22] Turunen HT, Sipila P, Pujianto DA, Damdimopoulos AE, Bjorkgren I (2011). Members of the murine Pate family are predominantly expressed in the epididymis in a segment-specific fashion and regulated by androgens and other testicular factors.. Reprod Biol Endocrinol.

[23] Fry BG, Wuster W, Kini RM, Brusic V, Khan A (2003). Molecular evolution and phylogeny of elapid snake venom three-finger toxins.. Journal of Molecular Evolution.

[24] Ploug M, Ellis V (1994). Structure-function relationships in the receptor for urokinase-type plasminogen activator. Comparison to other members of the Ly-6 family and snake venom alpha-neurotoxins.. FEBS Letters.

[25] Ploug M (2003). Structure-function relationships in the interaction between the urokinase-type plasminogen activator and its receptor.. Current Pharmaceutical Design.

[26] Coronel CE, Winnica DE, Novella ML, Lardy HA (1992). Purification, structure, and characterization of caltrin proteins from seminal vesicle of the rat and mouse.. Journal of Biological Chemistry.

[27] Yenugu S, Richardson RT, Sivashanmugam P, Wang Z, O'Rand MG (2004). Antimicrobial activity of human EPPIN, an androgen-regulated, sperm-bound protein with a whey acidic protein motif.. Biol Reprod.

[28] Rajesh A, Madhubabu G, Yenugu S (2011). Identification and characterization of Wfdc gene expression in the male reproductive tract of the rat.. Molecular Reproduction and Development.

[29] Cornwall GA, Hann SR (1995). Specialized gene expression in the epididymis.. Journal of Andrology.

[30] Hinton BT, Lan ZJ, Rudolph DB, Labus JC, Lye RJ (1998). Testicular regulation of epididymal gene expression.. J Reprod Fertil Suppl.

[31] Metayer S, Dacheux F, Dacheux JL, Gatti JL (2002). Comparison, characterization, and identification of proteases and protease inhibitors in epididymal fluids of domestic mammals. Matrix metalloproteinases are major fluid gelatinases.. Biol Reprod.

[32] Torres AM, Kini RM, Selvanayagam N, Kuchel PW (2001). NMR structure of bucandin, a neurotoxin from the venom of the Malayan krait (Bungarus candidus).. Biochemical Journal.

[33] Zhou CX, Zhang YL, Xiao L, Zheng M, Leung KM (2004). An epididymis-specific beta-defensin is important for the initiation of sperm maturation.. Nat Cell Biol.

[34] Liu Q, Hamil KG, Sivashanmugam P, Grossman G, Soundararajan R (2001). Primate epididymis-specific proteins: characterization of ESC42, a novel protein containing a trefoil-like motif in monkey and human. Endocrinology. 142: 4529-4539.. Endocrinology.

[35] Rodriguez CM, Kirby JL, Hinton BT (2001). Regulation of gene transcription in the epididymis.. Reproduction.

[36] Charest NJ, Petrusz P, Ordronneau P, Joseph DR, Wilson EM (1989). Developmental expression of an androgen-regulated epididymal protein.. Endocrinology.

[37] Nayfeh SN, Barefoot SW, Baggett B (1966). Metabolism of progesterone by rat testicular homogenates. II. Changes with age.. Endocrinology.

[38] Harris ME, Bartke A (1974). Concentration of testosterone in testis fluid of the rat.. Endocrinology.

[39] Harris ME, Bartke A (1981). Androgen levels in the rete testis fluid during sexual development.. Experientia.

[40] Kaplan N, Morpurgo N, Linial M (2007). Novel families of toxin-like peptides in insects and mammals: a computational approach.. Journal of Molecular Biology.

[41] Yenugu S, Hamil KG, Birse CE, Ruben SM, French FS (2003). Antibacterial properties of the sperm-binding proteins and peptides of human epididymis 2 (HE2) family; salt sensitivity, structural dependence and their interaction with outer and cytoplasmic membranes of *Escherichia coli*.. Biochem J.

[42] Rodrigues A, Queiroz DB, Honda L, Silva EJ, Hall SH (2008). Activation of toll-like receptor 4 (TLR4) by in vivo and in vitro exposure of rat epididymis to lipopolysaccharide from Escherichia Coli.. Biology of Reproduction.

[43] Biswas B, Yenugu S (2011). Antimicrobial responses in the male reproductive tract of lipopolysaccharide challenged rats.. American Journal of Reproductive Immunology.

