# Supplementary material for: Genomic Organization, Tissue Distribution and Functional Characterization of the Rat Pate Gene Cluster
Source: PLoS One. 2012 Mar 30;7(3):e32633. doi: 10.1371/journal.pone.0032633 (PMC3316536; doi:10.1371/journal.pone.0032633)
Supplement: Figure S1 — Alignment of rat Pate mRNA and chromosomal sequence. Bold upper case letters indicate exons and lower case letters indicate introns. Amino acids are indicated in bold single letters. Predicted signal peptide cleavage site is underlined and in italics. The conserved ten cysteines are indicated in blue and underlined. Numbers in parentheses indicate amino acids of the protein. (DOC) [file pone.0032633.s001.doc]

**Figure S1. Alignment of rat *Pate* mRNA and chromosomal sequence.**

***Pate-P***

1 gatttcactg caggatccgg aaaagacctg gaagcaccaa gggcgagatt aactctgact

61 catttttcct tgaatttaag ttttgcctaa atggaagttt tacagatgat cattttagca

121 ccattaagtt agtcaaaagt ctgtgctccc ctctggctat atgctcacct catttcctgg

181 tatcccactt gccactcttc ctatcctagg ctacaatata tacctgcctt cctgatatga

241 gtcctgagca ctctgtcttc tcctctgctg atttctggtc tgaagatcaa ttgcactgca

(20) ***M G K D I L L L L L G L S L L V S S M Q***

301 **ATGGGAAAGG ACATCTTGCT GCTCCTGCTG GGCCTGTCTT TGCTAGTGAG CTCCATGCA**a

361 ggtaagatcc tggcagacag cagtggttgg cactggtggg aaaacacact gatactacag

// 1081 tacaaacaac tcttttcctg gatattgctt cttcttttac cagagcaaca agttcagctc

1141 ccgttggtgc ttgattttgg atcctgaatg agacccttat actggctttt gtcttttcAG

(20) ***A L* T C I K C E R F N S Q G I C E R G E**

1201 **CTTTGACATG TATCAAGTGT GAAAGGTTCA ATTCTCAGGG GATTTGTGAG AGAGGAGAAG**

(18) **G C C E A Q P G E K C A S L I T Y K**

1261 **GTTGCTGTGA GGCTCAACCT GGTGAGAAAT GTGCCTCACT TATAACCTAT AAAG**gtatgt

1321 aagttcttag agctctgaaa gtcatctcgc ttgccctaca gtgatgaacc catctccagg

// 1681 tgcttatatt tcagatgtgc tttccatctg attctttact gactccccaa ctctttctac

(7) **D G K I Q F G**

1741 ggatatttac attttgcctc ttgactttct tcttctacag **ATGGCAAAAT TCAGTTTGGA**

(20) **S Q R C A D L C Y R G T V E N G G L T I**

1801 **AGCCAGAGAT GTGCTGATCT TTGCTACAGA GGGACTGTTG AGAATGGAGG TCTGACAATA**

(16) **K M N C C S H R S F C N K P Y P ***

1861 **AAAATGAACT GTTGCTCACA CAGGTCTTTC TGCAATAAAC CATATCCGTG A**tacttaatt

1921 gtactgttgg attttaggtg agttcatacg ctttccctgt gctcctgcaa tctcataact

1981 ctgggcagtc agttgccagc actacacaaa ctaacaactg tattcactta gcattatgtg

2041 ctgaagcatc aacagttcaa caggctagag agctctttca tgcttcccac aaaccctggt

2101 gctccctcat tttcacatca tggtttcctt cccttttccc ctatatccct caaacacaat

2161 agatatatgg cataagtttc ttcttctctt ccatctacac tgattcaaca aatacattct

2221 gactgtcaaa cacagcactg

***Pate-Q***

1 ttgaatttaa gttttcccta atgggaagct ttacagatga tcatttttag catgattaag

61 ttagtgaaaa tctgtgctcc cctctggcta cttgctcagt ccatttcctg gtatcccaca

121 tgctgttcat gctatcctag gctacaatat aaacctacct tcctgaatgg gtcctgagca

(3) ***M E K H***

181 tgctgtcctc tcccctgctg atttctcttc tgaagatcaa tctcactgca **ATGGAAAAGC**

(17)  ***I L L L L L G L S L L V S S*** ***L Q***

241 **ACATCTTGCT GCTCCTGCTG GGCCTGTCAT TGCTAGTGAG CTCCCTACAA G**gtaagatcc

301 tagcagatgg tagtgcttca cacaggtggg cagacacact gatactgcag attaggggac

// 1021 gctcttttac tggctattgt ttatttattt accagagcca caagctcagc acccattggt

(3) ***A* L T**

1081 gcttgagttt ggatcctgaa taagaccctt acactggatt tgtcttttca g**CTTTGACAT**

(20) **C I T C E R F N S R G I C E R G E G C C**

1141 **GTATCACGTG TGAAAGGTTC AATTCTCGGG GGATTTGTGA GAGAGGAGAA GGTTGCTGTC**

(15) **Q A Q P G E K C A S F I T Y K**

1201 **AGGCTCAACC TGGTGAGAAG TGTGCCTCAT TTATAACCTA TAA**aggtatg taaggtctgg

// 1621 ttctggttta ctttccatct gactctttac taactcacca actcttttca cagacattta

(10) **D G K V Q Y G S Q K**

1681 cattttgcct cttgactttc ttcttctac**A GATGGAAAAG TTCAGTATGG AAGCCAGAAA**

(20) **C A D V C F S G T V E N G G L T V R M N**

1741 **TGTGCTGATG TTTGCTTCAG TGGGACTGTT GAGAATGGAG GCCTGACAGT AAGAATGAAC**

(13) **C C S H R S F C N K A H T ***

1801 **TGTTGCTCAC ACAGGTCTTT CTGCAATAAA GCACATACCT GA**tacttaat tgtactgttg

1861 gatttttagg tgagtaccta ctccttccct gtgttcttgt aatctcataa ccctggacag

1921 tcagttgcca gcacgtgtat ccacttagca tgagctgctg atgttttaac agttcaacag

1981 gctagagaga tgttccctgc ttcccactag ccctggcact ccctcattgt tacatcatgg

2041 tttccttcat ttttccccta tgtccctcca acacaatagc tatatggcac aagttgcttc

***Pate-F***

1 gatattggca aaacacccat gaacaggtgt gtcaggaagc tcctgcccac atcccaggag

61 cagctcagag aagagtctac agtctgctct catctaccct gacccattct aggtacttgc

(7) ***M G K L L L L***

121 tgccagtgct ctgctgtgtc ttctgcaaac tcagactgag **ATGGGCAAGC TCCTGCTCCT**

(13) ***L L L L G A F V L L L*** ***I Q***

181 **GCTCCTGCTG CTAGGAGCTT TTGTTCTTCT GTTGATCCAA** ggtgagaatg agtcttcaga

241 ggctgaaaca ggggtgaagt caggagctcc acatagctga cagtaaactt aagatggtgg

// 8701 tcaggagtgc ccacttgatc aggcggtgag gtctctctcc cacggggttt gggagcagag

8761 agctgctgcc atgccttatt tcttaactgt tcttaccctt gttggtccta gctcaatcaa

(9) ***A* Q E R I C M S C**

8821 ccctggaatc gctcatttta tctctttttt cta**GCCCAAG AAAGAATATG CATGTCCTGC**

(20) **H M F V N G K C V E S E G K C T M E D G**

8881 **CACATGTTTG TGAATGGCAA GTGTGTAGAA AGTGAAGGTA AATGCACCAT GGAGGACGGC**

(15) **G A C R T R D I Y L F N A R G**

8941 **GGTGCATGCA GAACCAGGGA CATCTATCTT TTCAATGCAA GAGGT**aatgc taatatggaa

9001 tccacaaggg agtctttcaa ggaagtggtg tttcctgtgg aagagcaggc ttgtgtggat

9061 accaaagact ctcatacact gaatttcaaa tattattttc ttacattaaa tatcaatgtt

(14) **G G F L Y N H T M L E C S K S C K A**

9121 cttttcca**GG TGGGTTTCTC TACAACCACA CTATGCTGGA GTGTTCTAAA TCTTGTAAGG**

(20)  **S E E S Y F H L K I S T F C C K S Q D**  **F**

9181 **CTTCAGAAGA GAGTTATTTT CATCTAAAAA TTTCAACTTT TTGTTGCAAA AGTCAAGACT**

(8)  **C N K Y K G K ***

9241 **TCTGCAATAA ATATAAAGGA AAGTGA**acac atgtgctaat tgatgaccag ctccttcact

9301 tgcttcattt tcccttgtgt gcctgctgga tccccctcca tacacaatct gctgaagatg

***Pate-A***

1 agagtctgca acctgctctc atctaccctg acccctcctg agtacttgct accagctgtt

(10) ***M G K L L F L L L L***

61 ctcctgtgtc ttctggtagc ccagactgag **ATGGGCAAGC TCCTGTTCCT TCTCCTGCTC**

(11) ***L G S F A L V F I Q V***

121 **CTGGGGTCAT TTGCTCTTGT GTTCATCCAA G**gtaatagtg agtccttcga ggcaaaaaca

181 ggggaaagga cagggactag agatggctga gagtaacggc agatgacagc ctgaatagct

// 4741 tttaatactc tagcattctt agtcactgca atcattttca cgccctttac atgtattttt

(19) ***Q A* T V C M V C N S F K S G H C L A**

4801 cccag**TCCAA GCTACAGTGT GCATGGTTTG CAACTCTTTT AAAAGTGGAC ATTGTTTGGC**

(20) **G K S N C T T R Y K P G C R T R N F F L**

4861 **TGGCAAGAGC AACTGCACTA CAAGATACAA GCCTGGATGC AGAACCAGGA ATTTCTTCCT**

(5) **F S N T G**

4921 **ATTCTCCAAT ACAGG**taagg tgtttgaact agtctaaagg cataattgct gagaagtaga

4981 tgtactcctt ctttcaaaaa catgtttggt cctggggaga aagtcccctc acagtgaata

(5) **K W V H N**

5041 ccagtatatt ttggacacta aatataaatg cttctatgtt ctagg**CAAGT GGGTCCACAA**

(20) **H T E L D C H K A C L A E N M Y L G A L**

5101 **TCATACCGAA TTGGACTGCC ATAAGGCATG TCTGGCTGAA AATATGTATT TGGGAGCATT**

(20) **K I S T F C C K G E D F C N K Y H G Q V**

5161 **GAAGATATCT ACCTTTTGCT GCAAAGGTGA AGATTTCTGT AATAAATATC ATGGCCAAGT**

(6) **V K K K I Y ***

5221 **AGTGAAGAAG AAAATTTACT AA**ctactgac tccttcatca ttgtcactgt gtctcagttt

5281 cccatttcct cttcctagaa tttctcaaca attccctctg tacatttaac ttgaagtaaa

***Pate-C***

1 agcttacttt gagatttgct tctgagaaat ctaatacaca ctcttctttg tacatgcctg

61 catggattat aaattcacta gagactggtc ttttctctga gtcattgctt ctcatcagct

(7) ***M E N L L K L***

121 gactggtact aacacagctg gaagaatctg cttaggagaa **ATGGAAAACC TACTGAAGCT**

(10) ***C L F L L C F E T*** ***G***

181 **GTGCCTCTTT CTTCTCTGCT TTGAAACAGG** tattgcctgc aggttgctcc cttatctgct

241 gtacgaagaa aagtaaatta gctcttattc cccactcctc tgtctgcggt gttgggacat

301 gttgactgga gcatatctgt aagccaaaat attggtgcag ccaagctatc cagaggtcag

361 atgatctatg ttgtctttga gaaagaacag tgacagtgat gataccctcc tcctctcatc

(14) ***F* *P*** **V Q C V K C N S Y K N G**

421 agtctttaca ctttagg**CTT CCCTGTACAG TGTGTGAAAT GTAATTCTTA CAAGAACGGA**

(20) **K C A G S Q Q T C T T R A G E M C M I R**

481 **AAATGTGCTG GAAGTCAGCA GACGTGCACT ACAAGAGCTG GTGAAATGTG TATGATACGC**

(7) **R T W Y A S E I**

541 **AGAACCTGGT ATGCATCTGA AA**gtaagtat gtgttctctg ctctggagaa tatctatttc

601 tccttcagga ttctaagtac cctatctaag attctggact tgaagaaaaa gaatcaactg

// 1681 attctggctc ttccaaactc atatgagtgt gtgtgtgtgt gtgtgtgtgt gtgtgtgtgt

(2) **I**

1741 gtgtgtgtat acatacatat atatgtgtgt gtatattttt tttcttcttc cacag**TTAAT**

(20) **K L L H A E T T C M G S C K I E E K T S**

1801 **AAACTGCTAC ATGCTGAGAC TACGTGCATG GGATCCTGTA AAATAGAAG AAAAACGTCT**

(20) **G Y L T I H T Y C C D F T D F C N D I G**

1861 **GGATATCTAA CAATACATAC CTACTGCTGT GATTTTACAG ATTTCTGCAA TGACATCGGT**

(6) **F P I V M T ***

1921 **TTTCCAATCG TAATGACTTA G**cataagcta gtttcagtcg ttctcatcag tcctttcctt

1981 ttcatttggt ctaaattttc ttacattgac ctctattttt actctggatt atagattttt

2041 ctggtaatca gtattgtata agtcacagaa cccagggaca ttctttgctc ttctgaatgt

2101 atatttgatc atatagtcta attcttatgc ttggcatccc tagccttatt accacgagta

***Pate-E***

(11) ***M G N E P K L G I I L***

1 acctgctgca agaattagaa gatcctca**AT GGGGAATGAA CCAAAGCTGG GCATCATTCT**

(6) ***L L C M Q T A***

61 **GTTACTCTGT ATGCAAACTG** gtaaatattg ggaaaccggt ttcctagcag ctaaggaatc

121 agtttaactt gaactctgac atatttttag taaatctttt gttttggggc tctatgggct

// 601 tgctacctga aatatatctc tgtaaggtaa aaccttaatg aggtcaagca gcctgggagt

(5)  ***L A* L L**

661 ttgagtactt ctcttttctt tcaagtcttt ctctttattt cttgcag**CTT TAGCGCTTCT**

(20) **C R E C T S Y L H Q K C L H E M K T C T**

721 **GTGTAGAGAA TGCACATCCT ATTTACATCA GAAATGTCTA CATGAAATGA AAACATGCAC**

(18) **A K D G E S C L T V R V W N I P Y S E**

781 **AGCCAAAGAT GGTGAATCCT GTCTAACTGT TAGAGTGTGG AATATACCTT ACAGTG**gtaa

841 gtagtgtgtg aagttaatgt gggagggacg gtgcctcttt ctcttcaagg cctcaaattt

// 1081 gtttgtcatt taatatgatc ttggtttact cttacttgga tttgtggcct gcttcattca

(9)  **Q V P N E A Y S**

1141 aaacaatata ttaattctat ttttgtctcc cacag**AACAG GTACCAAATG AAGCATACTC**

(20) **R C Q K N C T T D E Y Y Y G D Y T V M I**

1201 **TAGATGCCAG AAAAACTGTA CAACAGATGA ATATTACTAT GGTGATTATA CAGTAATGAT**

(20) **K C C E A Y D F C N D L L V P I S E W S**

1261 **AAAATGTTGT GAGGCATATG ACTTTTGTAA TGATCTTCTT GTGCCAATTA GTGAATGGTC**

*****

1321 **CTGA**agaacc ttcagattat cttatggctt cctccagtct tcctcatctt

***Pate-N***

1 cctcattgtt cagagtctag gacattactc agagagtggg aggccagggg aaagtctcag

61 taacagctcc atctcacatc ctgtccagag aacagttcct gagcacttgc tggagactga

(7) ***M D W L L F L***

121 ctgtttatct catccctctt ttattgtttt cctctaacaa **ATGGACTGGC TCCTGTTTCT**

(15) ***L F P G L L I L Y K S H*** ***T M G***

181 **TTTGTTTCCA GGACTACTGA TTTTGTACAA GTCTCATACA ATGGG**tgagt gttatggcaa

241 gaaaacaaag aaggaagaga tctagggtat gggattgata taccaagcat catgatcaga

// 541 gtgaaatgag atctcctttt tcttgtcccc actgcatccc aggtcttttt tctcaacctt

(1) **E**

601 ctctttagca tcaatgccta ccccattgtt atgatgtact atctatgccc tatagg**GGAA**

(20) **P S F C T S C D E Y V D D T C R R N L G**

661 **CCCAGTTTTT GCACTTCCTG TGATGAATAT GTTGATGATA CCTGCAGAAG AAACTTGGGA**

(20) **V C H P R Y P D F A C Q T K E V Y I Q L**

721 **GTCTGCCATC CCAGATATCC TGACTTTGCC TGCCAAACCA AAGAAGTATA TATTCAACTT**

(3) **N T G**

781 **AACACTGGA**g gtgaggtgaa ggccaaggga ggggagagag actattttat gacttggttt

// 1141 tcaagctgga agggaaagaa tggaaaattc atctacatct cttctcattg gtaaagactg

(10) **E Y L Y K Y S I L G**

1201 tatatttatg tcactgccct actttcttta **GAATATCTGT ACAAATATTC TATATTGGGC**

(20) **C P R R C V E Y V R F I K F E K N I F S**

1261 **TGCCCAAGAA GATGTGTGGA ATACGTGCGC TTCATCAAGT TTGAGAAAAA CATCTTCTCC**

(20) **C C N E S Y C N S F Q A K H T H F K E N**

1321 **TGCTGCAATG AAAGCTACTG CAACAGTTTT CAAGCAAAAC ATACTCACTT CAAAGAAAAT**

(3) **N F V ***

1381 **AATTTTGTCT AA**gctcttcc tgttaggcac atcctgccca tttcttcctg aacaagcttc

1441 aatgtttgga agtttcttca ctcccatcct acacccacaa tctgctgaga gccaaagggc

***Pate***

1 actcatcact gtataaatag tggggcttcc tgtcctaacc tgaacctttc cacctccaaa

(17)***M F K P H L L R C L T L L C Y L R*** **V**

61 **ATGTTCAAGC CCCACTTACT GAGATGCCTG ACCTTACTCT GCTATTTGAG GG**gtgagtca

121 tgaagagtca ccagttggtg agctatgtcc atgatcaaag aatggtgaga actcccatga

// 301 agcagatggt ttgccaacag actgagcgat tcctagaagc tgtgaggtgg gggctggaaa

(4) ***F F G***

361 attatattct gcagatagta tgaattttac cttttggttt tttcaacag**T TTTCTTTGGG**

(8) **S L P G D A N K** **P**

421 **TCGCTTCCAG GTGATGCTAA CAAAC**gtgag tagaaatggt tgtgattaat ggctacaaag

481 atgggggagt tgagtggagc ttggtgggca ctggcagcta aaagcaaacc acagctaggt

961 caaagggagg gtaaacctca gacccaactg cacgattgtt ctctttctct ctcacctgaa

(11)  **D K V L I H E N N N**

1021 cag**CTGATAA AGTACTCATT CATGAAAATA ACAAT**ggtaa gtacaaaaag ccctagcctg

// 1201 tgctttgatg ggtgtggggg ttcaggacac tcccagctgt aagcaggatt cacacttgaa

(2) **V V**

1261 gtagaggata ggctctttgc aggtagtaac ttctcctctc ccacctctct tcaa**GTTGTG**

(20) **E I V Q C R M C H L Q F P G E K C S R G**

1321 **GAAATTGTGC AGTGCAGGAT GTGCCACCTC CAGTTCCCAG GAGAAAAGTG CAGCAGAGGT**

(19) **R G I C T A T V E E A C M A G K I F K K**

1381 **AGAGGAATAT GTACTGCAAC AGTAGAAGAG GCCTGCATGG CTGGAAAAAT CTTCAAAA**gt

1441 gagtggtggg atcaagaagc aaaaacagga aagagaaagt gggatgtgtg ggccttgagg

// 2041 tctgaatact ggagtatgaa ccacatgtgg tgatttttag ataccagtgt gcgttgggga

(1)

2101 gaagtagatt tacagaatcc tcttccaagg ttgctgatct gttccctttc ttctgcag**AG**

(20) **D G T M W L K F M G C L K N C A N V K K**

2161 **GATGGTACCA TGTGGTTAAA ATTCATGGGC TGCTTAAAGA ACTGTGCTAA TGTGAAAAAA**

(20) **I K W G S Y L V D F R C C R G H D M C N**

2221 **ATAAAATGGG GCAGCTATCT GGTGGACTTC AGATGCTGCC GGGGCCACGA CATGTGCAAT**

(3) **E R F ***

2281 **GAAAGGTTTT AG**acacttct gtctttctgg gctcccacca tgtgtgtgat gggatggcct

2341 cttacaaaga cctccggagt ttaactctgc agaaaaggac tgcaaacaca tggctctgcc

***Pate-alternate transcript***

1 gatctattct tttttttttt ttaacggttc acagacttta ttggtttaac aatgaacagc

// 361 cctagactca tcactgtata aatagtgggg cttcctgtcc taacctgaac ctttccacct

(17) ***M F K P H L L R C L T L L C Y L R V***

421 ccaaa**ATGTT CAAGCCCCAC TTACTGAGAT GCCTGACCTT ACTCTGCTAT TTGAGGG**gtg

// 661 gaggaagcag atggtttgcc aacagactga gcgattccta gaagctgtga ggtgggggct

(2) ***F F***

721 ggaaaattat attctgcaga tagtatgaat tttacctttt ggttttttca acag**TTTTCT**

(10)  ***G*** **S L P G D A N K P**

781 **TTGGGTCGCT TCCAGGTGAT GCTAACAAAC** gtgagtagaa atggttgtga ttaatggcta

// 1261 tggtaggaaa ggctcaaaaa gaatccaaga taaaaggcaa aatgagccct gagggaagag

1321 aggtgcaaag ggagggtaaa cctcagaccc aactgcacga ttgttctctt tctctctcac

(11)  **D K V L I H E N N N V**

1381 ctgaacag**CT GATAAAGTAC TCATTCATGA AAATAACAAT G**gtaagtaca aaaagcccta

1621 ttgaagtaga ggataggctc tttgcaggta gtaacttctc ctctcccacc tctcttcaag

(20)  **V E I V Q C R M C H L Q F P G E K C S** **R**

1681 **TTGTGGAAAT TGTGCAGTGC AGGATGTGCC ACCTCCAGTT CCCAGGAGAA AAGTGCAGCA**

(20) **G R G I C T A T V E E A C M A G K I F** **K**

1741 **GAGGTAGAGG AATATGTACT GCAACAGTAG AAGAGGCCTG CATGGCTGGA AAAATCTTCA**

(1) **T**

1801 **AAA**gtgagtg gtgggatcaa gaagcaaaaa caggaaagag aaagtgggat gtgtgggcct

// 2281 ctctcacagt ggtaatccca gatgcatcta agcctctcat gatggccttt ctacctctag

(6) **S S F Q S** **F**

2341 taaaatctta aaaccactca ccatttattt tcttttgggt ag**CCTCCAGC TTCCAATCCT**

(20)  **T S E Y W S M N H M W ***

2401 **TCACATCTGA ATACTGGAGT ATGAACCACA TGTGGTGATT TTTAGATACC AGTGTGCGTT**

2461 **GGGGAGAAGT AGATTTACAG AATCCTCTTC CAAGGTTGCT GATCTGTTCC CTTTCTTCTG**

2521 **CAGAGGATGG TACCATGTGG TTAAAATTCA TGGGCTGCTT AAAGAACTGT GCTAATGTGA**

2581 **AAAAAATAAA ATGGGGCAGC TATCTGGTGG ACTTCAGATG CTGCCGGGGC CACGACATGT**

2641 **GCAATGAAAG GTTTTAG**aca cttctgtctt tctgggctcc caccatgtgt gtgatgggat

***Pate-2***

1 gatcctggga actggaagta ctgttgttct gaaaaattta tgatgtaatt tagatgaata

61 gcccaaggac aggtcaccgt ttcctttcaa gtgagaaaag agccagtgcc ttcctcagga

(7) ***M L V I V C L***

121 agctgttgaa gacaaggctt gcactatgtt tgctctgttc **ATGCTGGTGA TAGTCTGTTT**

(6) ***F C Q Y W G***

181 **GTTCTGCCAG TATTGGGGT**a agtcctggtg ataaaggtac tcaagagtgg cccaatgctg

241 gggaaggagg ggtttccaaa ctggctgtct gagattgtaa caaaggaaac tgcttttcct

(7) ***V L N* E P E K D**

301 tctcttccca ggt**GTCCTTA ATGAACCTGA AAAAG**gtacg atcatggctg gcataaagtc

// 781 tattctggga atgttatgac ccacagatca atagtaactt acagagaaag ctctaattgc

(3)  **L G**

841 agacgttttc tccagactgc attcactaag tacttctttc tgtgtctagt ag**ATCTTGGA**

(20) **T M C Y K C K K Y H L G L C Y G L M R S**

901 **ACAATGTGTT ATAAATGTAA GAAATATCAT CTTGGGTTAT GCTACGGACT CATGAGATCC**

(20) **C T L K H R Q S C A A E N F Y I L T N R**

961 **TGCACACTGA AGCATAGACA GTCCTGTGCT GCTGAGAACT TTTACATACT CACGAACAGA**

(1) **G**

1021 **GG**taacgtgg ggaatgggat gatgggcagc taaccagacc ttcccttcta aacagcaggg

// 1201 atcagttatg gagtacagga aattatggaa ttttgaggat aggttgttac cagaaacatg

(2) **Q S M**

1261 agagtgagca ataaattctt caaagttctt tcctctttca ctttctcagg **GCAGAGCATG**

(20) **Y H Y S R L S C M T N C E D I N F L S F**

1321 **TATCATTATT CAAGACTGTC ATGTATGACC AACTGTGAGG ACATCAACTT CCTGAGTTTT**

(20) **E R R T E L I C C K H S S Y C N L P M G**

1381 **GAAAGGAGGA CAGAGCTAAT TTGTTGCAAG CACAGTAGCT ATTGCAACCT CCCAATGGGA**

(1) **L ***

1441 **CTCTAG**ttct gaatttatta tgggtatggt atcattcttc aacttactac caactccctt

1501 ttccccaaag tttgtattta ctctccccac taactaacaa taaatgggaa aggcatttgt

1561 ccatgaaaag agaatcagtc atatgagaaa ctgggctggg agtttgtcct

***Pate-2 alternate transcript***

1 tggaaatagg aaccaatgtg atcaattagg atttaggaat atatcctgag ggagtcaaac

61 acgatgtctt gatcctggga actggaagta ctgttgttct gaaaaattta tgatgtaatt

121 tagatgaata gcccaaggac aggtcaccgt ttcctttcaa gtgagaaaag agccagtgcc

(3) **M L V** **I**

181 ttcctcagga agctgttgaa gacaaggctt gcactatgtt tgctctgttc **ATGCTGGTGA**

(18)  **V C L F C Q Y W G K S W ***

241 **TAGTCTGTTT GTTCTGCCAG TATTGGGGTA AGTCCTGGTG** **ATAAAGGTAC TCAAGAGTGG**

301 **CCCAATGCTG GGGAAGGAGG GGTTTCCAAA CTGGCTGTCT GAGATTGTAA CAAAGGAAAC**

361 **TGCTTTTCCT TCTCTTCCCA GGTGTCCTTA ATGAACCTGA** aaaaggtacg atcatggctg

421 gcataaagtc tttggttcat ttgtgacacc ttcaggagta aacctccctt ccatttactg

// 901 ctctaattgc agacgttttc tccagactgc attcactaag tacttctttc tgtgtctagt

961 a**GATCTTGGA ACAATGTGTT ATAAATGTAA GAAATATCAT CTTGGGTTAT GCTACGGACT**

1021 **CATGAGATCC TGCACACTGA AGCATAGACA GTCCTGTGCT GCTGAGAACT TTTACATACT**

1081 **CACGAACAGA GG**taacgtgg ggaatgggat gatgggcagc taaccagacc ttcccttcta

1141 aacagcaggg cctttcactc ttaggatgag tgggagagtg gagttgatgc agatgagcag

1201 tcttgcaaag actgcagaga tggatctttc cttaacatag cactgagtga tggaggggcg

1261 ggggtctctc atcagttatg gagtacagga aattatggaa ttttgaggat aggttgttac

1321 cagaaacatg agagtgagca ataaattctt caaagttctt tcctctttca ctttctcagg

1381 **GCAGAGCATG TATCATTATT CAAGACTGTC ATGTATGACC AACTGTGAGG ACATCAACTT**

1441 **CCTGAGTTTT GAAAGGAGGA CAGAGCTAAT TTGTTGCAAG CACAGTAGCT ATTGCAACCT**

1501 **CCCAATGGGA CTCTAG**ttct gaatttatta tgggtatggt atcattcttc aacttactac

1561 caactccctt ttccccaaag tttgtattta ctctccccac taactaacaa taaatgggaa

1621 aggcatttgt ccatgaaaag agaatcagtc atatgagaaa ctgggctggg agtttgtcct

1681 catttctaag caaatctttg ctaattcttt ttgtcatatc tagcagggca ttttgatctg

1741 tggacagtcc tggtcatcat gagtaggtcc agaattgatc acctcgtgta caagcccacg

1801 agaatttggc tcaaaatgtc atcccacctt tgtacaggga aatctgtaaa tatactctgt

1861 gttggtctgc accaagtgtt ctgagttggg

***Pate-Dj***

1 atgaataatc atcaactttt gctggaggag aaagcttgtc cctggagccc agcagactgg

61 gtctttgtta gctttcctgt ggaaggatta gctgtcagac ttgtgcttgc agcccttcct

(9) ***M N R H F L L L V S***

121 agctttgctc cacacaatac atctgggtcc aa**ATGAACAG GCACTTCTTG CTGCTCGTCT**

(7) ***L F C L I V E***

181 **CCCTCTTCTG CCTCATTGTG G**gtgagttcc agaggctcct ccctgaggag tgagcttcag

241 tgtcaggaac aagagaactg gaggggaatc ttcacacgta gagccatgtc catgtggact

// 1381 agctctttat ctgcctaccc acccttggaa gatcccttca cccagctttg ctatctgtct

(3) ***A T T***

1441 acaaattcca aatccatgtc ctcaagcgga gctgtgtgct ctctcataca g**AAGCAACAA**

(20) ***L* K C T T C H L R T Q S D H C R R G F** **G**

1501 **CACTGAAGTG TACAACATGC CACCTCCGCA CACAGTCAGA CCATTGCAGA AGAGGCTTTG**

(19)  **V C L A Q K H E T C M S L R I Y S N**

1561 **GTGTTTGCCT TGCTCAGAAG CACGAAACAT GCATGAGCTT GAGGATCTAC TCCA**gtaagt

1621 gtcagggttg ggtgcagtca tgtgaagcat gggcaacttc ttaaaaaggg ctatggcaga

// 1921 gggtgctctt gggaaggaga agctgggaag tcatccataa acctacctgg ttaaggcaat

1981 gagtgttctg ccttggatac tgagatcact gccttaccac tgcttctttc ttattgtag**A**

(20) **G S L Q T S Y M V C Q R F C K N L A Y N**

2041 **TGGCTCTCTC CAGACCTCAT ACATGGTGTG TCAGAGATTC TGCAAGAATT TGGCATACAA**

(20) **F N N R T Y I H K C C N Y D F C N F R L**

2101 **CTTCAACAAT CGAACTTATA TTCATAAATG TTGCAACTAT GATTTTTGTA ACTTCAGACT**

*****

2161 **TTAA**gatact ccccccaacc tcattctaga aggtcccaca tgtttttctt cttttacact

2221 ctgtttatcc tgcctttcca ccctgtatac atcgaggatg ctcctgctct atccccatca

2281 ttcatcctga ttcctttaat ttggatctca
