# Supplementary material for: Genomic Organization, Tissue Distribution and Functional Characterization of the Rat Pate Gene Cluster
Source: PLoS One. 2012 Mar 30;7(3):e32633. doi: 10.1371/journal.pone.0032633 (PMC3316536; doi:10.1371/journal.pone.0032633)
Supplement: Figure S2 — Three dimensional structures of rat PATE and PATE-F. The ten conserved cysteines are shown. Pairs of cysteines that may take part in disulfide bonding are shown in same color. (PPT) [file pone.0032633.s002.ppt]

## Slide 1
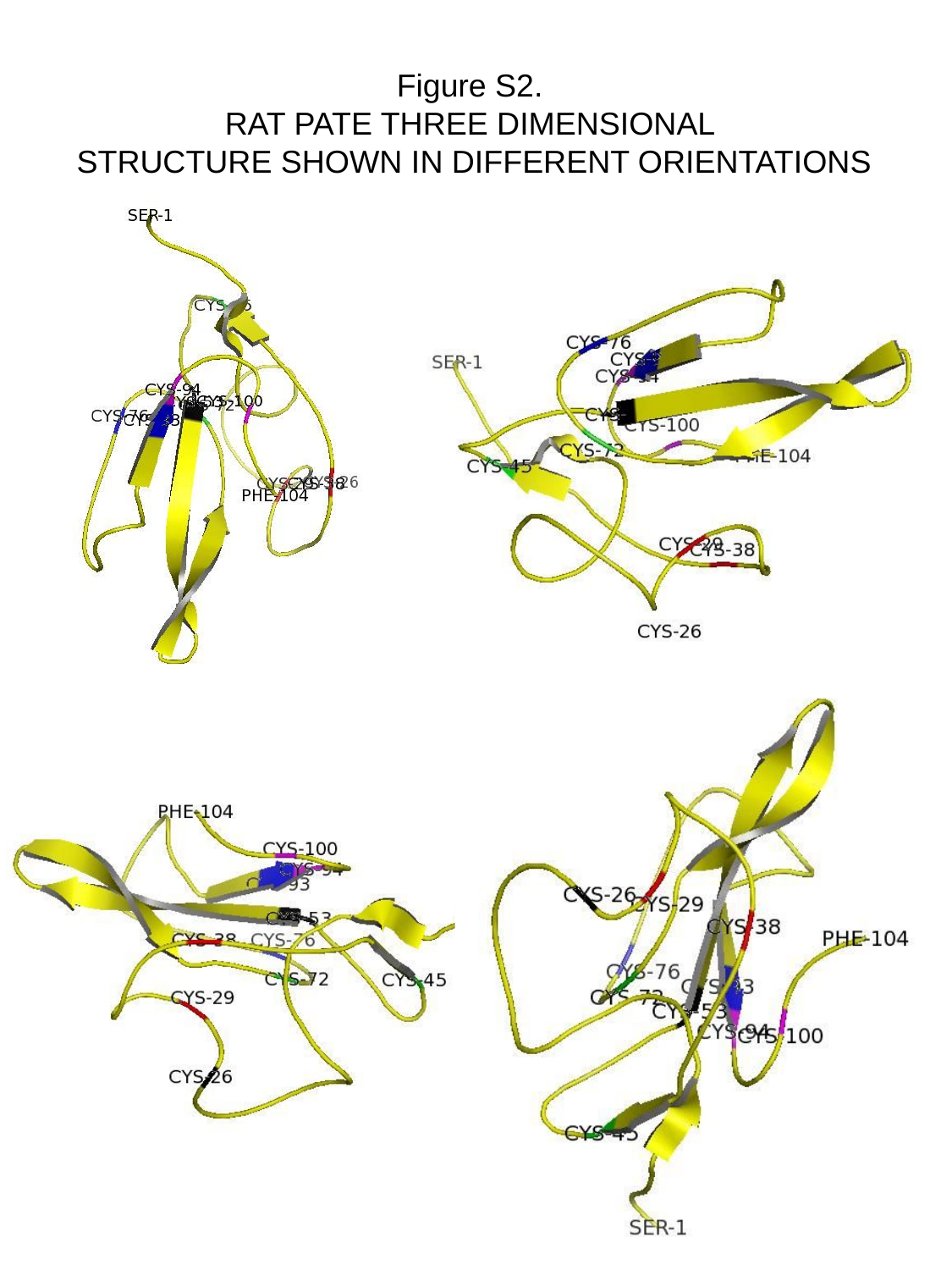

Figure S2.
RAT PATE THREE DIMENSIONAL
STRUCTURE SHOWN IN DIFFERENT ORIENTATIONS

## Slide 2
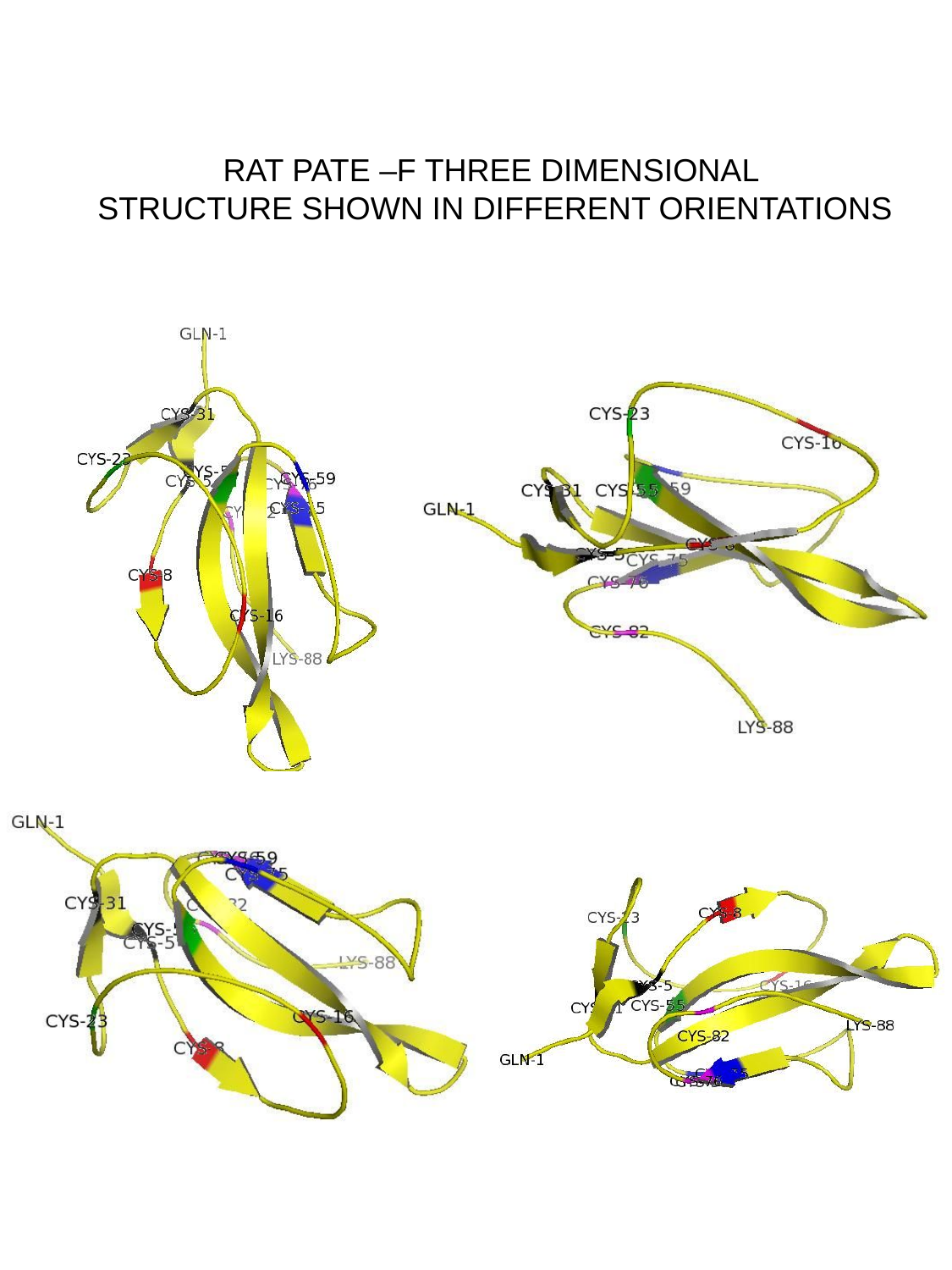

RAT PATE –F THREE DIMENSIONAL
STRUCTURE SHOWN IN DIFFERENT ORIENTATIONS
