# Supplementary material for: Genomic Organization, Tissue Distribution and Functional Characterization of the Rat Pate Gene Cluster
Source: PLoS One. 2012 Mar 30;7(3):e32633. doi: 10.1371/journal.pone.0032633 (PMC3316536; doi:10.1371/journal.pone.0032633)
Supplement: Figure S3 — Manual alignment of PATE, PATE-F and Bucandin. Cysteines in same color may form disulphide bonds in PATE and PATE-F similar to that of Bucandin. (DOC) [file pone.0032633.s003.doc]

**Figure S3. Alignment of mature PATE, PATE-F and Bucandin.**

PATE-F --------------QER-------I**C**MS**C**HMFVN-GK**C**VESEGK**C**TMED-GGA**C**RTRDIY 37

PATE SLPGDANKPDKVLIHENNNVVEIVQ**C**RM**C**HLQFPGEK**C**SRGRGI**C**TATV-EEA**C**MAGKIF 59

BUCANDIN -----------------------ME**C**YR**C**GVS-G---**C**HLKI-T**C**SAEE—-TF**C**Y----- 23

PATE-F LFNARGGGFLYNHTMLE**C**SKS**C**KASEESYFHLKISTF-**CC**KSQDF**C**NKYKGK 88

PATE ----KKDGTMW-LKFMG**C**LKN**C**ANVKKIKWGSYLVDFR**CC**RGHDM**C**NERF-- 104

BUCANDIN ----KWLNKISNERWLG**C**AKT**C**TEIDT--WN--VYNK-**CC**TTN-L**C**NT---- 63
