# Supplementary figures and images for: Genomic Organization, Tissue Distribution and Functional Characterization of the Rat Pate Gene Cluster
Source: PLoS One. 2012 Mar 30;7(3):e32633. doi: 10.1371/journal.pone.0032633 (PMC3316536; doi:10.1371/journal.pone.0032633)

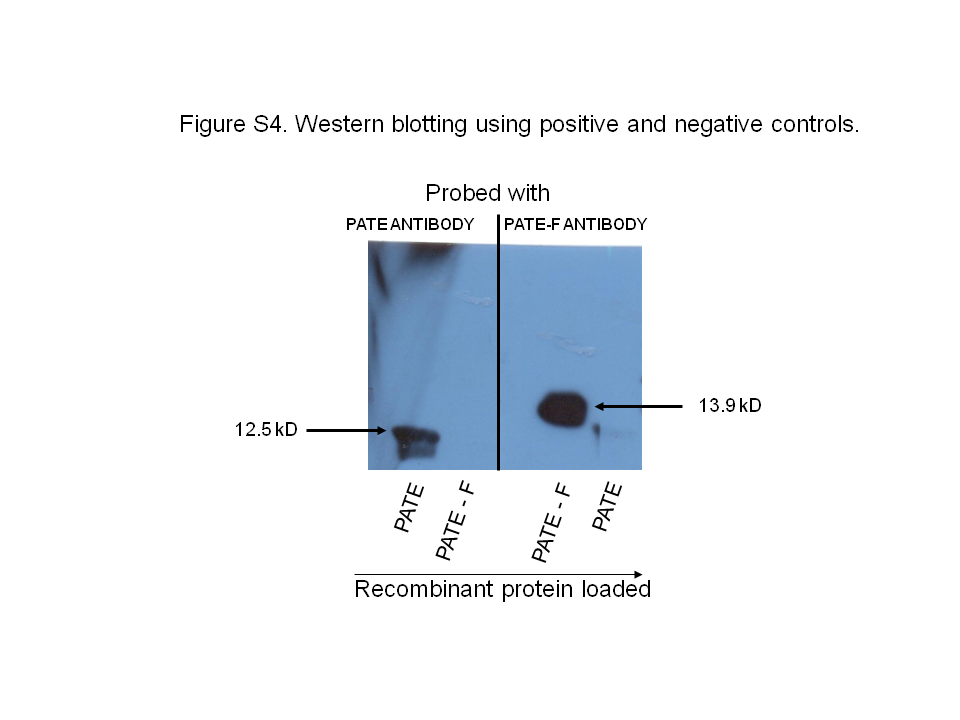

Supplement: Figure S4 — Western blotting using positive and negative controls. PATE and PATE-F recombinant proteins were probed with the polyclonal antibodies. For PATE, PATE-F was used as a negative control and vice-versa. (TIF) [file pone.0032633.s004.tif]
